# A quantitative LC-MS/MS method for residues analysis of 62 plant growth regulators and the investigation of their effects on the quality of traditional Chinese medicinal: *Codonopsis Radix* as a case study

**DOI:** 10.3389/fchem.2025.1587915

**Published:** 2025-07-15

**Authors:** Hongxu Zhou, Yue Ren, Yangli Wang, Jing Su, Xiangmin Zhou, Siyu Huang, Rui Yan, Jun Zeng, Min Chen, En Zhang, Xiaohu Chen

**Affiliations:** ^1^ CQMPA Key Laboratory for Quality Control and Evaluation of Traditional Chinese Medicine, Chongqing Institute for Food and Drug Control, Chongqing, China; ^2^ Chongqing Key Laboratory of New Drug Screening from Traditional Chinese Medicine, Integrative Science Center of Germplasm Creation in Western China (Chongqing) Science City and Southwest University, SWU-TAAHC Medicinal Plant Joint R&D Centre, College of Pharmaceutical Sciences, Southwest University, Chongqing, China; ^3^ School of Pharmacy, Chengdu University of Traditional Chinese Medicine, Chengdu, China

**Keywords:** plant growth Regulators, determination method, *Codonopsis Radix*, pesticide residue, untargeted plant metabolomics

## Abstract

Plant growth regulators (PGRs) enhance the biosynthesis of plant secondary metabolites but can cause environmental pollution and health risks, especially if synthetic or overused. Here, we developed a simple, high-throughput method using salting-out extraction and LC-MS/MS to analyze 62 PGR residues. The extraction, chromatographic conditions, and spectrometric parameters were systematically optimized. The extraction process was performed with acetonitrile-water (1:1), EN15662 extraction salt and d-SPE sorbent. This method was applied to analyze commercial and field trial *Codonopsis Radix* (CR) samples. The limit of quantification (LOQ), for 62 PGRs ranged from 0.03 to 82.50 μg/kg, and the limit of detection (LOD) ranged from 0.01 to 18.58 μg/kg. Furthermore, we employed plant metabolomics to assess changes in secondary metabolites in CR following fertilizer application and conducted a correlation analysis to explore the relationship between PGRs and secondary metabolites. In commercial samples, residues of 10 PGRs were detected, while in field trial samples, residues of 7 PGRs were found. In plant metabolomics, the arrangement of CR samples, which have been exposed to different fertilization levels, along the axes of partial least squares-discriminant analysis (PLS-DA) indicates that the chemical composition of CR experiences substantial alterations once a particular fertilization threshold is surpassed. The correlation analysis showed that PGRs boost amino acid metabolite synthesis and inhibit alkaloid biosynthesis. This study focuses on quality and safety concerns from the unchecked use of PGRs in CR production. It offers a framework for standardized cultivation and quality control to ensure the sustainable development of Traditional Chinese Medicine.

## 1 Introduction

Plant growth regulators (PGRs), such as chlormequat chloride (CCC), gibberellic acid (GA), ethephon (ET) and abscisic acid (ABA), are synthetic or natural substances used to manipulate plant growth, including hormones and other compounds (Santner et al., 2009). Plant hormones are natural substances produced by plants that regulate growth and development, such as auxins, gibberellins, and cytokinins. Essentially, all plant hormones can be considered PGRs, but not all growth regulators are hormones. In the field of agriculture, the application of PGRs has the potential to significantly enhance crop yield through the promotion of root development, the regulation of flowering, and the control of fruit maturation (Pu et al., 2018; Todeschini et al., 2018). Additionally, these regulators can improve the quality of produce by augmenting size, enhancing color, and extending the shelf life of fruits and vegetables. The widespread utilization of PGRs has demonstrated significant potential for enhancing agricultural productivity and generating substantial economic benefits. However, similar to other synthetic pesticides, PGRs are not without drawbacks; they contribute to environmental pollution and pose health risks, some of which may be irreversible. PGRs can elicit both acute and chronic toxic effects, impacting various physiological functions contingent upon factors such as dosage, exposure routes, duration of exposure, and the developmental stage of the organism (Huang et al., 2022; Hussein et al., 2015; Li et al., 2021). Reproductive and developmental disorders represent the primary adverse effects associated with PGRs (Wang and Hao, 2023). Globally, the incidence of infertility is on the rise, a phenomenon that can be partially attributed to both direct and indirect exposure to environmental pesticides (Le et al., 2020). Hence, it is of great practical significance to develop an effective and high-throughput method for the analysis of PGR residues.

In the trace analysis of multi-residue PGRs, it is challenging to mitigate matrix effects arising from the presence of numerous active components in the complex matrix, as well as interferences resulting from the diverse physicochemical properties of various PGRs. Consequently, the optimized analytical conditions required for the detection of acidic PGRs (such as auxins and their analogues), basic PGRs (such as cytokinins and their analogues), hydrophilic PGRs (such as quaternary ammonium plant growth inhibitors), and lipophilic PGRs (such as azole plant growth inhibitors) differ substantially from one another. Furthermore, the intricate composition of constituents in traditional Chinese medicines often poses challenges to the extraction and isolation processes of target analytes. Consequently, up to date, few methods have been reported to simultaneous determination of multi PGRs residues in Chinese medicines. Chayanis et al. established a method for determination of 39 PGRs root and rhizome Chinese medicines, such as *Panax* ginseng and *Codonopsis* Radix (Sutcharitchan et al., 2020). Wei et al. established LC-MS/MS methods for simultaneous determination of 23 PGRs, respectively, in root and rhizome Chinese medicines (WEI, 2017). Zhang et al. combined CZIF-8/CS-MS with UPLC-MS/MS to effectively analyze 4 PGRs in *Schisandra chinensis*, showing its potential for routine monitoring of low PGR levels in agricultural products (Zhang et al., 2024). Zhao et al. developed a liquid chromatography-tandem mass spectrometry (LC-MS/MS) method for the quantification of eleven plant growth retardants, facilitating the effective analysis of these compounds in *Ophiopogon japonicus* (Zhao et al., 2018). Nevertheless, the above detection methods exhibit various limitations, including inadequate sensitivity, a restricted linearity range, and the necessity for meticulous control of experimental variables, among others.

*Codonopsis Radix* (CR), a traditional tonic medicinal material in China name Dangshen, is one of valuable medicine food homology plants and has been widely used as a traditional Chinese medicine and a functional food for more than 2000 years in China. The 2020 edition of Chinese Pharmacopeia Commission has officially recorded the dried root of *Codonopsis pilosula* (Franch.) Nannf., *C. pilosula* (Franch.) Nannf. var. modesta (Nannf.) L.T.Shen, or *C. tangshen* Oliv. As a cheap substitute of ginseng, CR has been consumed as a dual-use with both botanical medicine and food application. The secondary metabolites from CR, such as alkaloids, phenylpropanoids, polyacetylenes, organic acids, and nucleoside, have been shown to have various physiological activities, including immune enhancement, antioxidative, antibacterial, anticancer, and anti-inflammatory (Dong et al., 2023; Liang et al., 2024; Shi et al., 2024). The rising demand for medicine has led to the excessive and improper use of fertilizers in cultivating CR, such as Zhuanggenling and Dangshen Qifei. This practice reduces the plant’s active ingredients, compromising its quality, and leaves toxic residues that harm the environment. PGRs are a category of synthetically produced compounds that modulate plant growth and development, and they are classified as a type of pesticide. When used rationally, PGRs have the potential to stabilize crop yields, enhance quality, and bolster resistance to environmental stressors. With the development of agriculture, the production and varieties of PGRs are increasing, PGRs have facilitated their extensive application in routine cultivation practices. This frequently leads to indiscriminate or excessive utilization, which negatively impacts the quality and safety of traditional Chinese medicinal materials and presents potential health risks to the public. Research has demonstrated that various PGRs, when applied individually or in combination, can substantially affect the accumulation of secondary metabolites, including terpenes, coumarins, flavonoids, isoflavones, and alkaloids. Specifically, research indicates that CCC has the capacity to upregulate lignin in *Scutellaria baicalensis* and saponins in *Bupleurum chinense*, while concurrently downregulating flavonoid levels in both *S. baicalensis* and *Lonicera japonica* (Qin et al., 2023). Furthermore, high concentrations of GA can reduce the levels of psoralen and isopsoralen in *Angelica dahurica* (Hou et al., 2013). As the diversity of PGRs continues to expand, there remains a significant deficiency in comprehensive residue testing for traditional Chinese medicinal materials, coupled with an absence of established maximum residue limits.

In this study, a comprehensive investigation was conducted concerning the residual presence of PGRs in CR following its application, along with its impact on the herb’s specialized metabolites. Firstly, ultra-high performance liquid chromatography (UPLC) and triple-quadrupole mass spectrometry method for determination of 62 PGRs was developed. The study examined factors influencing sample preparation, including extraction pH, acetonitrile-water ratio, extraction method and time, and various cleanup sorbents. Chromatographic and spectrometric parameters were optimized for effective separation, sensitivity, and specificity of the target analytes in the sample matrix. Next, the established method was applied in the analysis of commercial product and field trial CR samples. Furthermore, we used plant metabolomics to analyze metabolites changes in CR after fertilizer application, identifying shifts in secondary metabolites. Finally, a correlation analysis was conducted to examine the intrinsic relationship between PGRs and the secondary metabolites of CR. The goals of the present study were: 1) to develop a method for the determination of PGRs multi-residues in CR. 2) to evaluate the influence of PGRs on comprehensive constituents of CR on untargeted plant metabolomics.

## 2 Materials and methods

### 2.1 Chemicals and reagents

For the source of all crtified reference standards of PGRs see Supplementary data. Commercial PGR product “Dangshenqifei” (including 4-NP, CCC and 2-Pyridylpropanol) were purchased from Yishun AIB Company (Gansu, China). Arginine, phenylalanine, proline, adenosine, tryptophan, and lobetyolin were purchased from National Institutes for Food and Drug Control (NIFDC, Beijing, China).

LC-MS grade ammonium formate and formic acid were purchased from Merk (Darmstadt, Germany). LC-MS grade acetonitrile and methanol were obtained from Fisher Chemical (CA, United States). Deionized water was produced by Milli-Q system (Milford, United States). The QuEChERS commercial products used for sample extraction and clean-up procedures were purchased from Dikma Technologies (Tianjin, China). Anhydrous magnesium sulphate, ammonium chloride, and ammonium acetate were purchased from Chron Chemicals (Sichuan, China).

### 2.2 Preparation of standard solutions

Stock solutions of individual PGR (0.1 mg/mL) were prepared by dissolving acetonitrile, and stored at −20°C. The intermediate standard mixture (0.1 mg/mL) was prepared with acetonitrile. Matrix-matched calibration standards of 1, 2, 5, 10, 25, 50, 100, 200, 400 ng/mL were prepared by means of serial dilution of the intermediate mixture with matrix extracts, respectively. All solutions were filtered through a 0.22 μm membrane prior to instrumental analysis.

### 2.3 Field trials and sample collections

Field trials were conducted on two cultivation bases located in Pinshun (E113^。^23′50, N36^。^59′54) and Huguan (E113^。^24′12,N35^。^59′12), Shanxi Province, China, respectively. These locations were selected due to their close geographical proximity and similar soil properties, which help to reduce variability in environmental conditions that could affect metabolomic profiles. Regarding plant genetic uniformity, we ensured consistency by using the same batch of seedlings for transplantation across all experimental plots, thereby minimizing genetic variation among the CR plants studied. There were 3 rows (Row 1‒Row 3) and 4 lines (Line 1-Line 4) on the trial plot (Supplementary Figure S1). Each plot was 3 m long and 2 m wide and separated by 0.5 m-distance, respectively. The one-year-old plants seedlings which had a diameter of 0.3–0.6 cm were obtained from a commercial nursery for transplanting in March 2020. In budding stage, the treatments started. Each plot was randomly treated with different doses and times (in June 2020). The spraying concentrations of Dangshenqifei solutions were 180 g a.i. hm^−2^ (the interval between treatments was 7 days). The Dangshens were classified into four groups according to different, no treatments (control), one time treatment (low dose), two times treatments (middle dose), and three times treatments (high dose) (Pérez-Jiménez et al., 2015). Fresh root samples were collected and processed immediately after harvesting (in September 2020). The samples included control groups (HG01-09 and PS01-09), low dose groups (HG10-18 and PS10-18), middle dose groups (HG19-27 and PS19-27), and high dose groups (HG28-36 and PS28-36). The randomly selected samples were immediately dried at 60°C and stored at −20°C. The origin, batch number, and fertilization mode of the herb medicine are displayed in Supplementary Table S1. A total of 72 batches of fresh samples were purchased from the two bases for LC-HR/MS metabolomics study.

In addition, 102 batches of CR samples were collected from manufacturers, pharmacies, hospitals, and supermarkets in China for PGRs residue determination study (Supplementary Table S5).

### 2.4 Preparation of sample

#### 2.4.1 Preparation of sample solution for PGRs residue determination

All 174 CR decoction pieces were pulverized and passed through 0.25 mm sieve prior to further preparation. The dry CR powder of 3 g was accurately weighed, and 15 mL of water were subsequently added to a 50 mL polypropylene centrifuge tube. The mixture was vortexed until the powder was completely soaked, then 25 mL of acetonitrile were added. The sample was shaken vigorously by an automated shaker (KS-260, IKA-Werke GmbH and Co. KG, Staufen, Germany) at 3000 times/min for 5 min. One pack of QuEChERS EN15662 extraction salt, containing 4 g MgSO4, 1 g NaCl, 1 g Na_3_Cit·2H_2_O, and 0.5 g Na_2_HCit·1.5H_2_O, was added into the sample tube. The tube was subjected to vigorous agitation using a shaker for 5 min, followed by cooling in an ice bath for 10 min. Subsequent to centrifugation at 4,000 rpm for 5 min, the supernatant was filtered through a 0.22 μm membrane prior to instrumental analysis. For the preparation of matrix-matched calibration standards, the standard mixture was incorporated into the extract before the addition of acetonitrile. All reference chemicals were dissolved in acetonitrile at a concentration of 1.0 mg/mL to serve as stock solutions. These stock solutions remained stable for at least 1 week at room temperature. A specified volume of the aforementioned stock solutions was combined and diluted to achieve the desired concentration for the standard stock solution. These standards remained stable for a minimum of 2 weeks when stored at 4°C.

#### 2.4.2 Preparation of sample solution for metabolite profiling

The dry powder (1 g) of each of 72 samples were extracted with 25 mL of 50% methanol in an ultrasonic bath for 60 min. The obtained extract was centrifuged at 14,000 rpm for 5 min. Subsequently, the supernatant was filtered through a 0.22 μm membrane filter prior to analysis. The test solution was then stored at 4°C. To assess the stability of the analytical system, a quality control (QC) sample was prepared by pooling equal volumes of the test solution from each batch of material.

### 2.5 Instrument and optimized analytic procedure

#### 2.5.1 Liquid chromatography triple-quadrupole mass spectrometry analysis for PGRs residue determination

An Agilent 1290 Infinity II LC System coupled with an Agilent 6470 triple quadrupole mass spectrometer was used (Agilent Technologies, Waldbronn, Germany). Separation was done using an ACQUITY UPLC CORTECS C18 (150 × 2.1 mm, 1.7 μm) from Waters. The mobile phase consisted of acetonitrile (solvent A: 0.05% formic acid, 5 mM ammonium formate) and water (solvent B: 0.05% formic acid, 5 mM ammonium formate). The mobile phase composition changed during the run as follows: 0–1 min, 10% A; 1.0–5.0 min, 10%–34% A; 5.0–8.0 min, 34%–37% A; 8.0–8.5 min, 37%–45% A; 8.5–15.0 min, 45%–75%; 15.0–15.1 min, 75%–100% A; 15.1–20.0 min, 100% A. The flow rate was set to 0.3 mL/min, the column temperature was set at 40°C, and the injection volume was 2 μL.

The ESI source operation parameters were as follows: Gas temp 300°C, Gas flow 5 L/min, Nebulizer 45 psi, Sheath gas temp 275°C, Sheath gas flow 11 L/min, Capillary −2.5 kV and +3.5 kV, Nozzle Voltage ±500 V. Quantification was performed using MRM. The retention time, quantitative and confirmative mass transitions, fragmentor, and collision energy for each analyte are shown in Table 1.

**TABLE 1:** Mass spectrometric parameters for 62 PGRs.

NO	Compounds	Precursor ion (*m/z*)	Product ion (*m/z*)	RT (min)	Fragmentor (V)	CE (V)	Polarity
1	CCC	122.1	63.1	1.12	110	25	Positive
		122.1	59.2	1.12	110	21	Positive
		122.1	58.2	1.12	110	37	Positive
2	Pix	114.1	98.1	1.13	110	33	Positive
		114.1	70.2	1.13	110	45	Positive
		114.1	58.2	1.13	110	33	Positive
3	2-Pyridylpropanol	138.1	120.1	1.14	80	17	Positive
		138.1	92.1	1.14	80	25	Positive
		138.1	65.1	1.14	80	41	Positive
4	DMASA	161.1	143.1	1.18	80	9	Positive
		161.1	115.8	1.18	80	17	Positive
		161.1	61.2	1.18	80	13	Positive
5	KT	216.1	148.0	4.70	80	13	Positive
		216.1	81.1	4.70	80	21	Positive
		216.1	53.2	4.70	80	53	Positive
6	ThiBZ	202.1	175	4.71	110	29	Positive
		202.1	131.1	4.71	110	41	Positive
		202.1	65.1	4.71	110	53	Positive
7	2iP	204.13	136.0	5.89	80	17	Positive
		204.13	119.0	5.89	80	41	Positive
		204.13	69.2	5.89	80	21	Positive
8	GA3	345.1	227.1	6.00	200	29	Negative
		345.1	143.1	6.00	200	37	Negative
9	6-BA	226.1	91.1	6.29	110	29	Positive
		226.1	65.1	6.29	110	60	Positive
		226.1	63.1	6.29	110	60	Positive
10	Dikegulac	275.1	217.1	6.37	80	5	Positive
		275.1	69.1	6.37	80	25	Positive
		275.1	59.2	6.37	80	25	Positive
11	4-FPA	169.0	125.1	6.65	80	9	Negative
		169.0	111.0	6.65	80	17	Negative
		169.0	91.1	6.65	80	37	Negative
12	Prohexadione	211.1	167.1	7.05	110	13	Negative
		211.1	123.1	7.05	110	13	Negative
		211.1	111.0	7.05	110	25	Negative
13	IAA	176.15	130.4	7.10	100	16	Positive
		176.15	103.1	7.10	100	28	Positive
14	DA-6	216.2	143.1	7.16	80	17	Positive
		216.2	100.1	7.16	80	17	Positive
		216.2	71.2	7.16	80	29	Positive
15	4-NP	138.0	108.0	7.25	110	17	Negative
		138.0	92.1	7.25	110	25	Negative
16	5-NG	168.0	153.0	7.51	80	13	Negative
		168.0	123.0	7.51	80	21	Negative
17	1-NAD	186.1	115.0	7.86	110	49	Positive
		186.1	141.1	7.86	110	21	Positive
18	ABA	263.1	219.1	7.96	140	13	Negative
		263.1	204.1	7.96	140	21	Negative
		263.1	153.1	7.96	140	9	Negative
19	Actidione	282.2	69.2	7.96	110	37	Positive
		282.2	57.1	7.96	110	33	Positive
		282.2	55.2	7.96	110	60	Positive
20	4-CPA	185.0	127.0	8.22	80	13	Negative
		185.0	111.1	8.22	80	9	Negative
21	IPA	190.1	130.1	8.42	80	17	Positive
		190.1	77.1	8.42	80	60	Positive
		190.1	55.2	8.42	80	21	Positive
22	TDZ	221.0	128.0	8.45	80	21	Positive
		221.0	102.0	8.45	80	17	Positive
		221.0	77.1	8.45	80	53	Positive
23	DCPTA	262.1	100.1	8.48	110	21	Positive
		262.1	72.2	8.48	110	29	Positive
		262.1	58.2	8.48	110	33	Positive
24	SMZ	202.1	104.0	8.52	110	29	Positive
		202.1	71.1	8.52	110	29	Positive
		202.1	68.1	8.52	110	41	Positive
25	Ancymidol	257.1	135.0	8.61	110	29	Positive
		257.1	81.1	8.61	110	33	Positive
		257.1	77.1	8.61	110	60	Positive
26	4-BPA	229.0	170.9	8.74	80	13	Negative
		229.0	79.0	8.74	80	45	Negative
27	IBA	204.1	186.1	9.54	80	13	Positive
		204.1	130.0	9.54	80	33	Positive
		204.1	117.0	9.54	80	37	Positive
28	4-IPA	276.9	218.9	9.72	110	13	Negative
		276.9	126.9	9.72	110	37	Negative
29	Cloprop	199.0	127.0	9.83	80	13	Negative
30	2-NOA	201.1	157.1	9.88	80	5	Negative
		201.1	143.1	9.88	80	17	Negative
		201.1	115.1	9.88	80	45	Negative
31	Mefluidide	309.0	176.1	9.96	140	25	Negative
		309.0	175.1	9.96	140	29	Negative
		309.0	133.0	9.96	140	25	Negative
32	2,4-D	219.0	161.0	10.36	110	13	Negative
		219.0	125.0	10.36	110	33	Negative
		219.0	89.1	10.36	110	41	Negative
33	SOK	202.1	145.0	10.76	50	9	Positive
		202.1	127.0	10.76	50	33	Positive
		202.1	115.0	10.76	50	49	Positive
34	ATZ	216.1	174.0	10.84	110	21	Positive
		216.1	104.0	10.84	110	33	Positive
		216.1	68.1	10.84	110	45	Positive
35	CPPU	248.1	129.0	10.96	80	21	Positive
		248.1	93.0	10.96	80	41	Positive
		248.1	66.1	10.96	80	60	Positive
36	GA7	329.1	267.1	11.12	200	13	Negative
		329.1	223.1	11.12	200	17	Negative
37	Benzanilide	198.1	105.0	11.29	110	21	Positive
		198.1	77.1	11.29	110	49	Positive
		198.1	51.1	11.29	110	60	Positive
38	GA4	331.1	257.1	11.32	200	25	Negative
		331.1	243.1	11.32	200	17	Negative
		331.1	213.1	11.32	200	33	Negative
39	DPU	213.1	94.1	11.63	110	21	Positive
		213.1	77.1	11.63	110	49	Positive
		213.1	51.2	11.63	110	60	Positive
40	Ethychlozate	237.0	191.0	11.8	110	17	Negative
		237.0	135.0	11.8	110	29	Negative
41	2,4,5-T	252.9	194.9	11.86	110	13	Negative
		252.9	158.9	11.86	110	33	Negative
42	Dichlorprop	233.0	161.0	11.92	80	13	Negative
		233.0	125.0	11.92	80	33	Negative
		233.0	89.1.0	11.92	80	45	Negative
43	TE	253.1	81.1	12.11	80	53	Positive
		253.1	69.1	12.11	80	25	Positive
44	TIBA	498.7	454.8	12.16	140	5	Negative
		498.7	126.9	12.16	140	25	Negative
45	Inabenfide	337.1	122	12.31	110	13	Negative
		337.1	78.1	12.31	110	37	Negative
46	Cyclanilide	272.0	228.0	12.44	80	9	Negative
		272.0	160.0	12.44	80	25	Negative
47	Flurprimidol	313.1	270.0	13.01	140	25	Positive
		313.1	269.0	13.01	140	41	Positive
		313.1	81.1	13.01	140	45	Positive
48	BR	481.4	315.4	13.25	110	32	Positive
		481.4	95.1	13.25	110	29	Positive
49	PHJ	255.2	177.0	13.4	110	5	Positive
		255.2	99.0	13.4	110	21	Positive
		255.2	81.0	13.4	110	60	Positive
50	PBZ	294.1	70.1	13.87	110	21	Positive
51	UCZ	292.1	125.0	13.87	140	33	Positive
		292.1	70.1	13.87	140	29	Positive
		292.1	57.2	13.87	140	37	Positive
52	TPN	264.2	70.1	14.21	110	29	Positive
		264.2	67.1	14.21	110	41	Positive
		264.2	55.2	14.21	110	53	Positive
53	CIPC	214.1	172.0	14.32	80	9	Positive
		214.1	154.0	14.32	80	21	Positive
		214.1	93.0	14.32	80	37	Positive
54	TBZ	308.2	125.0	14.46	110	49	Positive
		308.2	70.1	14.46	110	25	Positive
		308.2	59.2	14.46	110	53	Positive
55	Chlorphonium chloride	361.2	159	14.95	110	49	Positive
		361.2	76.1	14.95	110	45	Positive
		361.2	57.2	14.95	110	57	Positive
56	Ethy 1-naphthaleneacetate	215.1	141.0	14.97	110	9	Positive
		215.1	115.0	14.97	110	57	Positive
57	Diniconazole	326.1	158.9	15.24	110	41	Positive
		326.1	70.1	15.24	110	37	Positive
		326.1	57.2	15.24	110	33	Positive
58	Pyraflufen-ethyl	413.0	339.0	16.27	110	21	Positive
		413.0	289.0	16.27	110	33	Positive
		413.0	253.0	16.27	110	45	Positive
59	Pyribenzoxin	610.2	413.1	17.85	80	5	Positive
		610.2	180.1	17.85	80	45	Positive
		610.2	77.1	17.85	80	60	Positive
60	Flumetralin	422.1	143.0	18.04	110	45	Positive
		422.1	108.2	18.04	110	57	Positive
		422.1	107.0	18.04	110	60	Positive
61	Butralin	296.2	240.1	18.22	80	13	Positive
		296.2	222.2	18.22	80	25	Positive
		296.2	57.4	18.22	80	29	Positive
62	DEF	315.1	168.9	18.86	140	13	Positive
		315.1	112.9	18.86	140	25	Positive
		315.1	57.2	18.86	140	29	Positive
63	ATZ-d_5_ (IS)	221.1	179.0	10.79	110	21	Positive
		221.1	101.1	10.79	110	29	Positive
		221.1	69.1	10.79	110	49	Positive

#### 2.5.2 Liquid chromatography quadrupole-orbitrap mass spectrometry analysis for metabolite profiling

The analyses were performed on an Ultimate 3000 UHPLC system coupled to Q-Exactive MS (Thermo Fisher Scientific, CA, United States). UHPLC analyses were performed on a Waters BEH C18 column (2.1 × 100 mm, 1.7 μm). The mobile phase was consisted of 0.1% formic acid in acetonitrile (A) and 0.1% formic acid in water (B), and the elution gradient profile was set as follows: 0–1.0 min, 3% A; 1.0–6.0 min, 3%–10% A; 6.0–8.0 min, 10%–20% A; 8.0–14.0 min, 20%–37% A, 14.0–20.0 min, 37%–90% A, 20.0–22.0 min, 90% A, 22.0–22.1 min 90%–5% A, and 22.1–25.0 min, 5% A. The flow rate was set at 0.3 mL/min, and the column temperature at 35°C.

The MS spectrometry analysis was carried out on HR-ESI-orbitrap under the conditions: full MS/ddMS^2^ mode; evaporation temperature, 350°C; capillary temperature, 320°C; spray voltage, 3.0 kV for negative ion mode and 3.5 kV for positive ion mode; aux gas flow rate, 10 arb; sheath gas rate, 35 arb; mass range (*m/z*), 100–1500.

A resolution of 70,000 was set for the Orbitrap analyzer. AGC target and maximum injection time were 3e^6^ and 100 ms, respectively. An inclusion-list-induced data-dependent MS^2^ scan method was developed to acquire MS/MS information. AGC target and maximum injection time were 1e^5^ and 50 ms, respectively. The MS^2^ scan resolution was 17,500. The stepped normalized collision energies (NCE) were used at the settings of 30%, 40%, and 50%. An Xcalibur 3.1 software (Thermo Fisher Scientific, San Jose, CA, United States) was used to control data acquisition.

### 2.6 Optimization of chromatographic separation and MS/MS condition for PGRs residue determination

Chromatographic separation is of paramount importance when measuring target compounds with diverse chemical properties, such as polarity and pKa, within a single analytical run. In this study, three columns were evaluated: Waters ACQUITY UPLC CORTECS C18 (150 × 2.1 mm, 1.7 μm), Luna Omega C18 (2.1 mm × 100 mm, 1.6 μm), and Waters ACQUITY UPLC BEH C18 (2.1 mm × 100 mm, 1.7 μm). Four distinct mobile phase systems were tested: 0.05% formic acid (FA) in methanol-0.05% FA in water, 0.05% FA in acetonitrile-0.05% FA in water, 0.1% FA in acetonitrile-0.1% FA in water, and 0.05% FA in acetonitrile with 5 mM ammonium formate-0.05% FA in water. Finally, the transitions, fragmentor voltages and collision energy (CE) voltages were optimized for 62 PGRs using the MassHunter Workstation Optimizer 10.0.127 (Agilent Technologies, Santa Clara, CA, United States).

### 2.7 Optimization of sample preparation methodology

QuEChERS, an acronym for “Quick, Easy, Cheap, Effective, Rugged, and Safe,” represents a widely adopted sample preparation methodology in the field of analytical chemistry. This technique was devised to streamline the sample preparation process, thereby enhancing accessibility and efficiency while maintaining the integrity and accuracy of analytical results. The QuEChERS method comprises two principal stages: salting-out extraction and dispersive solid-phase extraction (d-SPE) cleanup. Salting-out extraction encompasses solvent extraction, liquid-liquid partitioning, and the incorporation of an extraction salt to facilitate the separation of the organic and aqueous phases. In this study, the QuEChERS technique was modified and optimized for the analysis of 62 PGRs in CR, detail in see Supplementary data.

### 2.8 Method validations for PGRs residue determination

The method validation was conducted following the guidance of SANTE/11312/2021 validation criteria for quantitative analytical methods: sensitivity/linearity, accuracy, precision, limit of quantification (LOQ), and specificity (Pihlström et al., 2021). The back-calculated concentrations of calibration standards should deviate no more than ±20% from the true concentrations using the relevant calibration curve. To determine the limit of detection (LOD), a signal-to-noise ratio (S/N) of 3 was utilized, while the LOQ was established at a signal-to-noise ratio of 10. The intra-day precision and accuracy were assessed by measuring QC samples (low, QCL; medium, QCM; high, QCH) within a single day. To evaluate the inter-day precision and accuracy, QC samples (QCL; QCM; QCH) were measured over three consecutive days. Acceptable mean recoveries (accuracy) were 70%–120%, with respective RSD (precision) < 20%. The LOQs were defined as the lowest spiking level of the validation meeting accuracy and precision criteria (n ≥ 5). Recovery was determined by calculating the corrected mean peak area ratio (analyte/IS) of each analyte spiked before extraction and dividing it by the corresponding ratio of each analyte spiked after extraction. This assessment was conducted at three QC concentrations (QCL; QCM; QCH) to ensure accuracy and reliability.

### 2.9 Statistical analysis

Statistical analysis and graphical representation were conducted using GraphPad Prism 9.5 software (GraphPad Prism Inc., CA, United States) and IBM SPSS Statistics 22. The raw LC-HRMS data files (.raw) were first processed using Compound Discover 3.2 (Thermo Fisher Scientific, CA, United States) for retention time alignment, peak picking, and annotation. The chromatographic peak data were normalized uniformly, and the multidimensional data were further analyzed by the SIMCA-*P* software 14.1 (Umetrics, Umea, Sweden) for multivariate data analysis by partial least squares-discriminant analysis (PLS-DA), orthogonal partial least squares-discriminant analysis (OPLS-DA). Multivariate statistical analysis was performed utilizing MetaboAnalyst 5.0 online platform (https://www.metaboanalyst.ca/). The significance level between the two groups was evaluated using either Student’s *t*-test or the Mann–Whitney test. Results were deemed statistically significant at *P* < 0.05.

## 3 Results and discussion

### 3.1 Optimization of LC-MS/MS conditions for PGRs residue determination

Initially, the selection of columns for the separation of PGRs was undertaken. Three conventional reverse-phase (RP) columns were evaluated. The results indicated that, in addition to offering comparable resolution and similar selectivity, the CORTECS C18 column produced superior peak shapes and higher signal intensity compared to the other columns tested. Subsequently, under the same elution procedure, different mobile phase systems were investigated. The results showed that acetonitrile-H_2_O (0.05% FA with 5 mM ammonium formate) mobile phase system showed higher column efficiency and better peak shape, besides comparable resolution. Therefore, CORTECS C18 column and mobile phase (0.05% FA in acetonitrile with 5 mM ammonium formate-0.05% FA in water with 5 mM ammonium formate) was used in the PGRs residue determination.

Optimization of MS parameters is another essential step for obtaining maximum sensitivity. The precursor ion was determined in scan with an *m/z* range of 50–750 followed by the optimization of MRM transitions and corresponding CE. The molecular ion [M + H]^+^ in positive mode was selected as the precursor ion for 38 PGRs, mostly basic compounds. The molecular ion [M - H]^-^ in negative mode was selected as the precursor ion for 19 PGRs, mostly acidic compounds. [M - Cl]^+^ ion was selected as precursor ion for 3 chloride salts,CCC, Pix, and Chlorphonium chloride. [M - Na]^-^ ion was selected as precursor ions for 2 sodium salts, 4-NP, and 5-NG. The optimized MRM transition and CE for each PGRs was listed in Table 1.

### 3.2 Optimization of sample preparation methodology

A QuEChERS method generally involves two steps: an extraction step based on partitioning via salting-out, followed by a purification step by using sorbents to remove matrix interferants. Several parameters, such as the extraction solvent, salt combination, sorbent type and amount, affect the sample preparation efficiency. The optimization experiments were evaluated by the recoveries using raw sample spiked with 100 mg/L of each PGR. Each experiment was conducted in 3 replicates.

#### 3.2.1 Extraction salt and pH

The pH value of the solution determines the state of the analytes. The salt addition can increase ionic strength and promote phase separation. The following nine combinations of solvent and salting-out agents based on the original, acidified, AOAC 2007 and EN15662 versions of QuEChERS were designed to investigated the extraction efficiency in different pH and salt environment: (A) original QuEChERS: ACN, 6 g MgSO_4_ + 1.5 g NaCl, (B) acidified QuEChERS: 1% acetic acid in ACN, 6 g MgSO_4_ + 1.5 g NaCl, (C) AOAC 2007: 1% acetic acid in ACN, 6 g MgSO_4_ + 1.5 g NaOAc, (D) EN15662: ACN, 4 g MgSO_4_ + 1 g NaCl +1 g Na_3_Cit·2H_2_O+ 0.5 g Na_2_HCit·1.5H_2_O, (E) NH_4_OAc: 1% acetic acid in ACN, 7.5 g NH_4_OAc, (F) MgSO_4_-NH_4_OAc: 1% acetic acid in ACN, 6 g MgSO_4_ +7.5 g NH_4_OAc, (G) NH_4_Cl: ACN, 7.5 g NH_4_Cl, (H) NH_4_O_2_CH: 1% acetic acid in ACN, 7.5 g NH_4_O_2_CH, (I) MgSO_4_-NH_4_O_2_CH: 1% acetic acid in ACN, 6 g MgSO_4_ +7.5 g NH_4_O_2_CH. Evaluated the recoveries of PGRs by using 15 mL of 0.1 ug/mL PGR water solution and 15 mL of ACN. The resulting recoveries of 39 basic and neutral PGRs were within 70%–120% for all nine versions of QuEChERS. As shown in Supplementary Figure S2, the satisfactory recoveries of 70%–120% of 23 acidic PGRs were obtained only among four QuEChERS versions (C, D, G, and I) which have the pH values (3–5) of both aqueous and acetonitrile layers. The presence of anhydrous MgSO_4_ significantly increased the distribution ratio of most acidic PGRs in organic layer by reducing amount of water. As a result, extraction salt (EN15662), 4 g MgSO4 + 1 g NaCl +1 g Na_3_Cit·2H_2_O+ 0.5 g Na_2_HCit·1.5H_2_O, was selected.

#### 3.2.2 Extraction solvent and time

Extraction efficiency is significantly affected by the solvent conditions. Water was used as pre-soaked solvent and added to dried pulverized samples in most method guideline for pesticide analysis (such as: EN15662, AOAC 2007). ACN extraction is commonly and widely used in QuEChERS method since it led to less interference. Different ratios and amount of acetonitrile-water in extraction solvent were investigated as followed: acetonitrile 15 mL, 5 mL H_2_O+ 10 mL (or 15 mL, 20 mL) acetonitrile, 10 mL H_2_O+ 10 mL (or 15 mL, 20 mL) acetonitrile, 15 mL H_2_O+ 15 mL acetonitrile. Mean analyte recoveries (n = 3) of PGRs in spiked samples (0.1 μg/g) were obtained by each investigated ratio.

Four highly polar compounds (CCC, Pix, DMASA, and 2-Pyridylpropanol) apparent increase in recoveries was observed with increasing ratio of acetonitrile-water in extraction solvent (Supplementary Figure S3). Maximum mean recoveries of the two PGRs were obtained by direct extraction of the samples with acetonitrile (Supplementary Figure S3). However, the poorest recoveries of 13 acidic PGRs (such as GA3, ABA, 2, 4, 5-T, etc.) were obtained with direct acetonitrile extraction. Dried pulverized samples should be pre-soaked with water prior to extraction with organic solvent to ensure adequate partitioning (Supplementary Figure S3). Since CR has strong water absorption, 3 g of pulverized CR occupied approximately 15 mL of water was opted for sample pretreatment. The mean recoveries of the target PGRs were showed within 70%–120% and no significant difference was found among different extraction methods employing different ratios and amount of acetonitrile-water (Supplementary Figure S3). Taken together, 15 mL H_2_O+ 15 mL acetonitrile was thus selected as extraction solvent to keep the procedure cheap and safe.

#### 3.2.3 Clean-up procedure

CR samples contain many components, such as glycosides, Polysaccharides, alkaloids, phenylpropanoids and organic acids (Dong et al., 2023). Therefore, effective purification is applied to reduce matrix effect and prevent the contamination of instrument. Different d-SPE sorbents play different roles. MgSO_4_ is used to eliminate co-extractives and residual water in the acetonitrile extract. C18 is used to remove nonpolar or medium-polar co-extracts such as lipids and sterols, while PSA is widely applied to adsorb polar impurities like organic acids, sugars and some pigments, and GCB has proved to remove nonpolar impurities and pigment compounds like carotenoids, chlorophyll, and sterols. Different combinations of C18, PSA and GCB are commonly used for purification in QuEChERS. Nine combinations of above the adsorbents were studied for the optimization of purification conditions: (C1) 400 mg PSA +1200 mg MgSO_4_; (C2) 400 mg PSA +400 mg C18 + 1200 mg MgSO_4_; (C3) 400 mg PSA +400 mg GCB +1200 mg MgSO_4_; (C4) 400 mg PSA +400 mg GCB +400 mg C18 + 1200 mg MgSO_4_; (C5) 400 mg PSA +45 mg GCB +400 mg C18 + 1200 mg MgSO_4_; (C6) 150 mg PSA +900 mg MgSO_4_; (C7) 150 mg PSA +150 mg C18 + 900 mg MgSO_4_; (C8) 150 mg PSA +45 mg GCB +900 mg MgSO_4_; (C9) 150 mg PSA +15 mg GCB +900 mg MgSO_4_;. As shown in Supplementary Figure S4, PSA exhibited a pronounced affinity for acidic and polar compounds. In the presence of PSA, the recoveries of 13 acidic plant growth regulators (PGRs), including 2,4-D, 2-NOA, and 4-CPA, were all below 30%. GCB demonstrated a strong binding effect toward planar compounds, such as DPU, ThiBZ, CPPU, inabenfide, and cyclanilide, resulting in recoveries lower than 20%. As previously mentioned, the advantages and disadvantages of each d-SPE sorbent in pesticide analysis have been extensively studied (Sutcharitchan et al., 2020). Therefore, 40 mg C18 + 10 mg PSA +10 mg GCB provided satisfactory recoveries (81.8%–106.2%) and thus, selected for optimum purification.

### 3.3 Quantitative method validation

#### 3.3.1 LOD, LOQ, and linearity

The LOQ for the 62 PGRs ranged from 0.03 to 82.50 μg/kg, while the LOD varied between 0.01 and 18.58 μg/kg, as detailed in Table 2. Linear regression analysis was conducted to ascertain the linear regression coefficients (*R* ^2^) by correlating the relative peak area (A_0_/Ai) with the concentrations of the standards. As presented in Table 2, the calibration curves for the 62 PGRs demonstrated strong linearity and satisfactory correlation coefficients (*R* ^2^ = 0.9902–0.9999).

**TABLE 2:** Standard curves, linear ranges, correlation coefficients, LODs and LOQs for 62 PGRs.

PGRs	Regression equation (weighting: 1/*x*)	*R* ^*2*^	Linear range (μg/kg)	LOD (μg/kg)	LOQ (μg/kg)
CCC	y = 0.369014 x + 0.001267	0.9998	1.00–400.00	0.17	0.55
PIX	y = 0.377926 x + 8.1910E-004	0.9999	10.00–4000.00	1.59	6.00
2-Pyridylpropanol	y = 0.359339 x + 0.002759	0.9976	10.00–4000.00	2.99	9.96
DMASA	y = 0.040146 x + 0.0024	0.9993	50.00–4000.00	11.80	39.34
KT	y = 1.111077 x + 0.005317	0.9997	1.00–400.00	0.01	0.04
ThiBZ	y = 0.986304 x – 3.6047E-004	0.9998	1.00–400.00	0.03	0.10
2iP	y = 1.087916 x + 0.002290	0.9999	1.00–400.00	0.18	0.60
GA3	y = 6.01154E-004 x −2.1079E-005	0.9983	50.00–4000.00	5.77	19.23
6-BA	y = 0.887078x – 4.6777E-004	0.9997	5.00–400.00	0.66	2.22
Dikegulac monohydrate	y = 0.023031 x + 1.4316E-004	0.9998	5.00–400.00	0.74	2.48
4-FPA	y = 0.002199 x + 6.3498E-006	0.9994	5.00–400.00	1.00	2.50
Prohexadione	y = 9.6076E-004 x – 1.4213E-005	0.9931	10.00–400.00	2.14	7.14
IAA	y = 1.5423E-02 x + 9.7701E-01	0.9955	5.00–400.0	1.72	2.00
DA-6	y = 0.583241 x – 0.001806	0.9990	1.00–400.00	0.07	0.24
4-NP	y = 0.036976 x + 0.005722	0.9998	1.00–400.00	0.32	0.50
5-NG	y = 0.001800 x – 2.5272E-005	0.9977	10.00–400.00	2.21	7.35
1-NAD	y = 0.646113 x + 0.003406	0.9994	1.00–400.00	0.12	0.38
ABA	y = 0.003860 x + 0.010818	0.9965	50.00–4000.00	13.91	20.00
Actidione	y = 0.001843 x + 0.003885	0.9950	10.00–4000.00	3.85	10.00
4-CPA	y = 0.002616 x + 2.5821E-005	0.9985	10.00–4000.00	1.70	4.19
IPA	y = 0.007952 x + 2.0101E-004	0.9989	10.00–4000.00	2.49	8.29
TDZ	y = 0.113377 x + 8.6829E-004	0.9994	10.00–4000.00	0.06	0.20
DCPTA	y = 0.738037 x + 0.002063	0.9998	1.00–400.00	0.07	0.22
SMZ	y = 0.227844 x + 0.001501	0.9983	1.00–400.00	0.12	0.40
Ancymidol	y = 0.174504 x + 0.001032	0.9985	5.00–400.00	0.41	1.35
4-BPA	y = 9.7408E-004 x – 1.7812E-005	0.9986	50.00–4000.00	5.36	17.86
IBA	y = 0.010039 x + 0.001050	0.9995	10.00–4000.00	2.12	7.05
4-IPA	y = 0.003826 x + 2.4884E-005	0.9995	5.00–400.00	0.80	2.66
Cloprop	y = 0.003535 x + 6.0894E-006	0.9994	10.00–4000.00	1.00	3.33
2-NOA	y = 0.002333 x + 1.0875E-005	0.9989	10.00–4000.00	1.21	4.03
Mefluidide	y = 0.010121 x + 3.3638E-004	0.9921	1.00–400.00	0.25	0.83
2,4-D	y = 0.003298 x + 4.8471E-005	0.9993	5.00–400.00	0.96	3.21
SOK	y = 0.153542 x + 5.6055E-004	0.9999	1.00–400.00	0.07	0.24
ATZ	y = 0.513334 x + 0.002656	0.9994	1.00–400.00	0.02	0.07
CPPU	y = 0.382229 x + 0.003996	0.9967	1.00–400.00	0.14	0.45
GA7	y = 0.005399 x – 7.6479E-005	0.9975	10.00–4000.00	1.40	4.67
Benzanilide	y = 0.860218 x + 0.001105	0.9999	1.00–400.00	0.09	0.29
GA4	y = 0.001175 x – 2.3011E-005	0.9920	10.00–4000.00	3.75	10.00
DPU	y = 1.530515 x + 0.011324	0.9991	5.00–400.00	0.56	1.86
Ethychlozate	y = 6.3597E-004 x + 3.5282E-006	0.9987	10.00–4000.00	2.98	10.00
2,4,5-T	y = 0.005728 x + 4.3953E-005	0.9977	10.00–4000.00	1.27	4.24
Dichlorprop	y = 0.007104 x + 9.6763E-006	0.9968	5.00–400.00	0.48	1.59
TE	y = 0.058487 x – 0.001652	0.9990	10.00–4000.00	5.00	10.00
TIBA	y = 0.001138 x – 1.0928E-005	0.9938	20.00–4000.00	10.00	20.00
Inabenfide	y = 0.026383 x + 5.5886E-004	0.9929	1.00–400.00	0.11	0.37
Cyclanliide	y = 0.030195 x + 5.8143E-004	0.9902	1.00–400.00	0.19	0.63
Flurprimidol	y = 0.124522 x + 2.7348E-004	0.9999	1.00–400.00	0.15	0.49
BR	y = 4.9091E-004 x + 7.8993E-006	0.9996	50.00–4000.00	7.50	24.04
PHJ	y = 5.74E-02 x + 2.37E-02	0.9956	100.00–4000.00	18.58	82.50
PBZ	y = 0.049898 x + 5.4315E-004	0.9963	10.00–4000.00	2.06	6.87
UCZ	y = 0.190379 x + 0.001806	0.9958	5.00–400.00	0.41	1.37
TPN	y = 0.520379 x + 0.003310	0.9993	1.00–400.00	0.05	0.18
CIPC	y = 0.001581 x + 4.2503E-005	0.9923	5.00–400.00	1.44	4.81
TBZ	y = 0.358111 x + 0.001784	0.9999	1.00–400.00	0.27	0.91
Chlorphonium chloride	y = 0.611617 x + 0.001088	0.9999	1.00–400.00	0.05	0.16
Ethy 1-naphthaleneacetate	y = 0.002666 x + 4.9059E-004	0.9909	10.00–4000.00	5.82	9.23
Diniconazole	y = 0.169462 x + 5.9229E-004	0.9999	5.00–400.00	0.48	1.59
Pyraflufen-ethyl	y = 0.064986 x + 3.3114E-004	0.9999	1.00–400.00	0.06	0.19
Pyribenzoxim	y = 0.653597 x + 0.008221	0.9968	1.00–400.00	0.01	0.03
Flumetralin	y = 0.001316 x + 7.2989E-005	0.9974	10.00–4000.00	4.98	10.00
Butralin	y = 0.052184 x + 2.4299E-004	0.9932	1.00–400.00	0.27	0.92
DEF	y = 0.222284 x + 0.001715	0.9997	10.0–4000.0	1.89	5.00

#### 3.3.2 Recovery, precision and accuracy

Three QC concentrations (QCL; QCM; QCH) of standard solutions were added in the CR sample to determine the recovery, which ranges from 62.46% to 129.04% (Supplementary Table S4), suggesting consistency and reproducibility of the recovery method. In this experiment, the intra-day and inter-day precision (RSD, %) ranged from 1.02% to 19.88% and 1.65%–19.99%, respectively (Supplementary Table S4).

### 3.4 PGRs residues in commercial products

**FIGURE 1:**
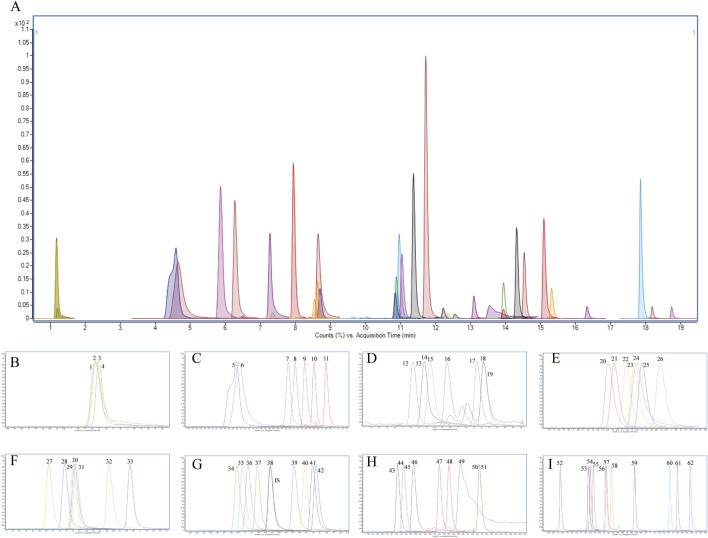
Extracted ion chromatogram (XIC) of **(A)** 62 PGRs in the standard mixture; **(B)** 1-4 corresponding to CCC, Pix, 2-Pyridylpropanol, DMASA, respectively; **(C)** 5-11 corresponding to KT, ThiBZ, 2iP, GA3, 6-BA, Dikegulac, respectively; **(D)** 12-19 corresponding to Prohexadione, IAA, DA-6, 4-NP, 5-NG, 1-NAD, ABA, Actidione, 4-FPA, respectively; **(E)** 20-26 corresponding to 4-CPA, IPA, TDZ, DCPTA, SMZ, Ancymidol, 4-BPA, respectively; **(F)** 27-33 corresponding to IBA, 4-IPA, Cloprop, 2-NOA, Mefluidide, 2,4-D, SOK, respectively; **(G)** 34-42 corresponding to ATZ, CPPU, GA7, Benzanilide, GA4, DPU, Ethychlozate, 2,4,5-T, Dichlorprop, respectively; **(H)** 43-51 corresponding to TE, TIBA, Inabenfide, Cyclanilide, Flurprimidol, BR, PHJ, PBZ, UCZ, respectively; **(I)** 52-62 corresponding to TPN, CIPC, TBZ, Chlorphonium chloride, Ethy 1-naphthaleneacetate, Diniconazole, Pyraflufen-ethyl, Pyribenzoxin, Flumetralin, Butralin, DEF, respectively. IS: ATZ-d_5_.

In 102 batches of commercial products CR, residues of ten PGRs were detected, as detailed in Figure 1 and Supplementary Table S5. These include CCC (1.67–454.82 μg/kg, 38 batches), IAA (42.51–1883.07 μg/kg, 10 batches), 4-NP (4.67–2949.33 μg/kg, 85 batches), Pix (2.72–747.91 μg/kg, 42 batches), UCZ (28.93–403.05 μg/kg, 17 batches), PBZ (6.24–991.81 μg/kg, 20 batches), 2-pyridylpropanol (10.85–914.49 μg/kg, 48 batches), actidione (12.84–70.36 μg/kg, 2 batches), indole-3-butyric acid (46.33–325.62 μg/kg, 5 batches) and ABA (100.53–655.89 μg/kg, 11 batches). Among these, IAA and ABA are endogenous PGRs. Among PGRs analyzed, 4-NP exhibited the highest detection rate at 83.33%, while actidione demonstrated the lowest detection rate at 1.96%, based on an examination of 102 batches of commercial products. 4-NP is a widely utilized plant growth regulator, known for its multifaceted roles in enhancing plant development. Its principal mechanism of action involves the regulation of plant hormone equilibrium, augmentation of plant stress resistance, improvement of photosynthetic efficiency, and stimulation of physiological processes such as cell division (Kazda et al., 2015; Szparaga et al., 2018).

### 3.5 PGRs residues in design of field trial

The PGRs residues detected in the specialized fertilizer used for CR in Huguan included CCC, 2-Pyridylpropanol, DCPTA, 2,4-D, 4-NP, 5-NG and other PGRs. Similarly, the specialized fertilizer utilized for CR in Pinshun contained residues of 2-Pyridylpropanol, DCPTA, 2,4-D, 4-NP, 5-NG and other PGRs. In 72 batches of CR from Huguan and Pingshun in Shanxi Province, residues of seven plant growth regulators were detected, as detailed in Supplementary Table S1. These include CCC (1.65–5.34 μg/kg, 12 batches), IAA (258.03–8305.30 μg/kg, 7 batches), 4-NP (2.41–50.97 μg/kg, 56 batches), 5-NG (26.16–313.81 μg/kg, 36 batches), 2,4-D (9.71–978.24 μg/kg, 21 batches), ABA (100.53–389.98 μg/kg, 5 batches) and 2-Pyridylpropanol (25.18–94.70 μg/kg, 19 batches). Among these, IAA and ABA are endogenous PGRs. Among PGRs, all CR samples were detected for CCC, 4-NP, 5-NG and 2-Pyridylpropanol. This indicates that the application of agricultural fertilizers can lead to residues of PGRs. Among the PGRs analyzed, post-fertilization treatment revealed a detection rate of 100% for 4-NP, 65% for 5-NG, and 26% for 2-pyridineethanol. CCC was identified in the agricultural fertilizer products of CR from Huguan, with a detection rate of 44% in the CR samples. In contrast, CCC was not detected in the agricultural fertilizer products from Pingshun, nor was it found in any CR samples from that region.

### 3.6 Metabolite variations of CR in different PGR fertilizer treatments

#### 3.6.1 Phytochemical analysis by UPLC-Q-orbitrap MS for CR

**FIGURE 2:**
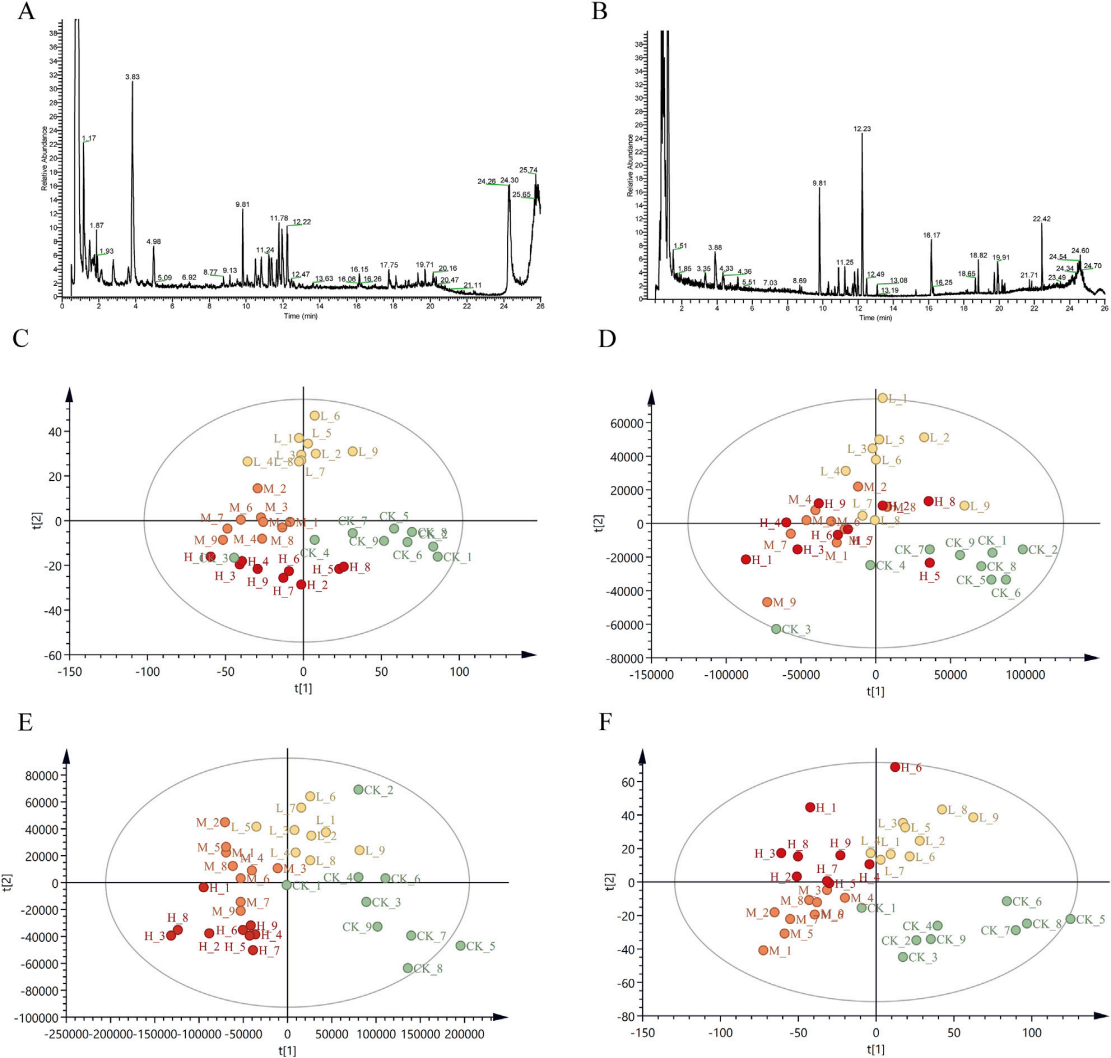
Total ion chromatograms (TIC) and PLS-DA score plot of CR in positive and negative ion mode **(A)** TIC of CR in positive ion mode; **(B)** TIC of CR in negative ion mode; **(C)** PLS-DA in positive mode for Pingshun; **(D)** PLS-DA in negative mode for Pingshun; **(E)** PLS-DA in positive mode for Huguan; **(F)** PLS-DA in negative mode for Huguan.

UHPLC-Q Exactive-MS/MS analysis was performed in both positive and negative ion modes to investigate the phytochemical composition of CR. The retention times, accurate molecular weights, quasi-molecular ions, molecular formulas, and MS/MS fragments observed in both ionization modes are summarized in Figures 2A,B and Supplementary Table S6. In the present study, a total of 61 compounds were identified, including 20 alkaloids, 11 organic acids, 10 polyacetylenes, 5 amino acids, 4 phenylpropanoids, 4 terpenoids, 3 phenylpropanoids, 1 nucleoside, and 3 other compounds, either unambiguously or tentatively.

#### 3.6.2 Methodology test

A methodological assessment was conducted on the QC samples to verify the accuracy of the metabolomics data. The QC sample solution was injected six times, and peak areas and retention times were recorded. The RSD values for ion retention times and peak areas were calculated, showing values below 1% and 7% (Supplementary Table S7), respectively, which indicates the instrument’s stability and precision. Six QC sample solutions were prepared in parallel, and the samples were subjected to continuous injection for subsequent analysis. The resulting data, comprising retention times and peak areas, were exported for further evaluation. RSD values were calculated, yielding results below 1% for retention times and below 7% for peak areas (Supplementary Table S7). These findings demonstrate that the analytical method exhibits robust repeatability. The same QC sample solution was injected and analyzed at six time points (0, 2, 4, 8, 12, 24 h) in the whole injection sequence, and peak areas and retention times were recorded. The RSD values for ion retention times and peak areas were calculated, showing values below 1% and 7%, respectively (Supplementary Table S7), indicating that the stability of data collection was very good.

#### 3.6.3 Multivariate statistical analysis

The metabolic data were analyzed using PLS-DA to explore the effect of PGR on metabolism in CR. The data of each group were classified via PLS-DA analysis, and the degree of aggregation and dispersion of samples could be observed. By analyzing the results of PLS-DA (Figure 2), it can be seen that PGR can change endogenous metabolites in CR. The PLS-DA model diagram illustrates the general trend of separation between untreated and variously fertilized CR samples. For PS CR in both positive and negative ion modes the distribution along the horizontal axis progresses from right to left in the following order: control group, low-dose group, middle-dose group, and high-dose group (Figures 2C,D). There is a partial overlap observed between the blank and low-dose groups, whereas the distinction between the middle-dose and high-dose groups is not pronounced (Figures 2C,D). However, a clear separation is evident between the control group and both the middle -dose and high-dose groups. For HG PLS-DA model are described as similar with that in PS (Figures 2E,F). To further validate the models, we performed random permutation tests with 200 iterations. The R2 and Q2 values of all original model were significantly higher than those of the permutation test (*p* < 0.001, CV-ANOVA), indicating that our PLS-DA model suffered from neither excessive randomness or overfitting (Supplementary Figures S5A–D). The distribution of CR samples, subjected to varying levels of fertilization, along the PLS-DA axes suggests that the chemical composition of CR undergoes significant changes following a specific threshold of fertilization treatment.

#### 3.6.4 Discovery of differential metabolites of CR in different PGR fertilizer treatments using a volcano plot and OPLS-DA modeling

**FIGURE 3:**
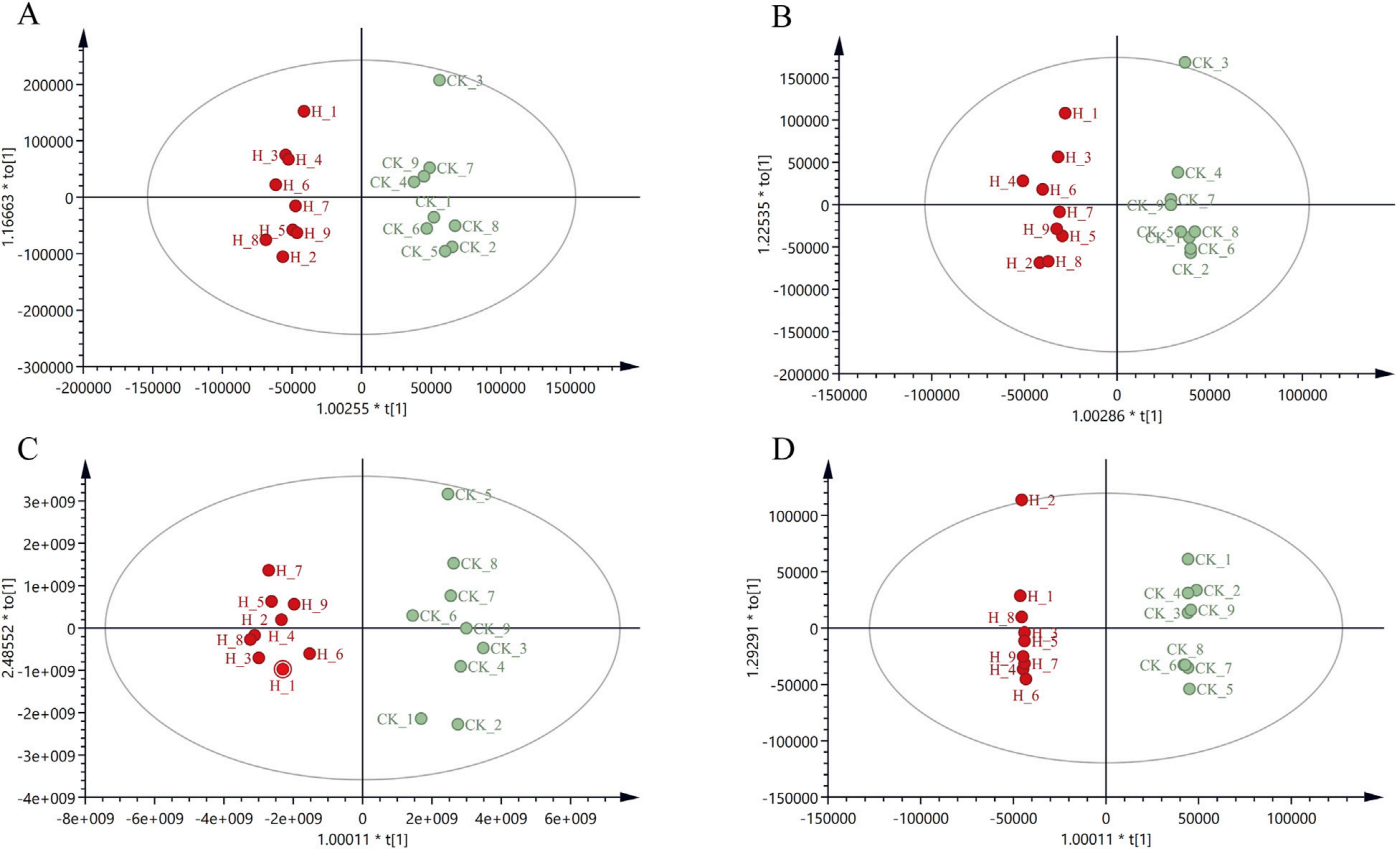
OPLS-DA score plot of CR in positive and negative ion mode for Pingshun and Huguan **(A)** OPLS-DA in positive mode for Pingshun; **(B)** OPLS-DA in negative mode for Pingshun; **(C)** OPLS-DA in positive mode for Huguan; **(D)** OPLS-DA in negative mode for Huguan.

To discover the candidate differential metabolites, we subsequently compared metabolite levels between the high-dose and control groups. To further elucidate which metabolites could effectively distinguish the high-dose group from the control group based on their differential expression, a supervised OPLS-DA was conducted on the CR metabolome profile (Figure 3). The results demonstrated a clear separation between the high-dose and control groups, indicating significant metabolite discrepancies as evidenced by the OPLS-DA. The parameters of OPLS-DA models including R2X = 0.646, R2Y = 0.998, Q2 = 0.648 in the ESI + mode, and R2X = 0.539, R2Y = 0.975, Q2 = 0.488 in the ESI- mode for Pingshun. The parameters of OPLS-DA models including R2X = 0.934, R2Y = 0.952, Q2 = 0.88 in the ESI + mode, and R2X = 0.627, R2Y = 0.999, Q2 = 0.847 in the ESI- mode for Huguan. All parameters are measures of the goodness of fit and prediction. Additionally, we conducted random permutation tests, which demonstrated that our OPLS-DA model was neither excessively random nor overfitted (Supplementary Figures S5E–H).

**FIGURE 4:**
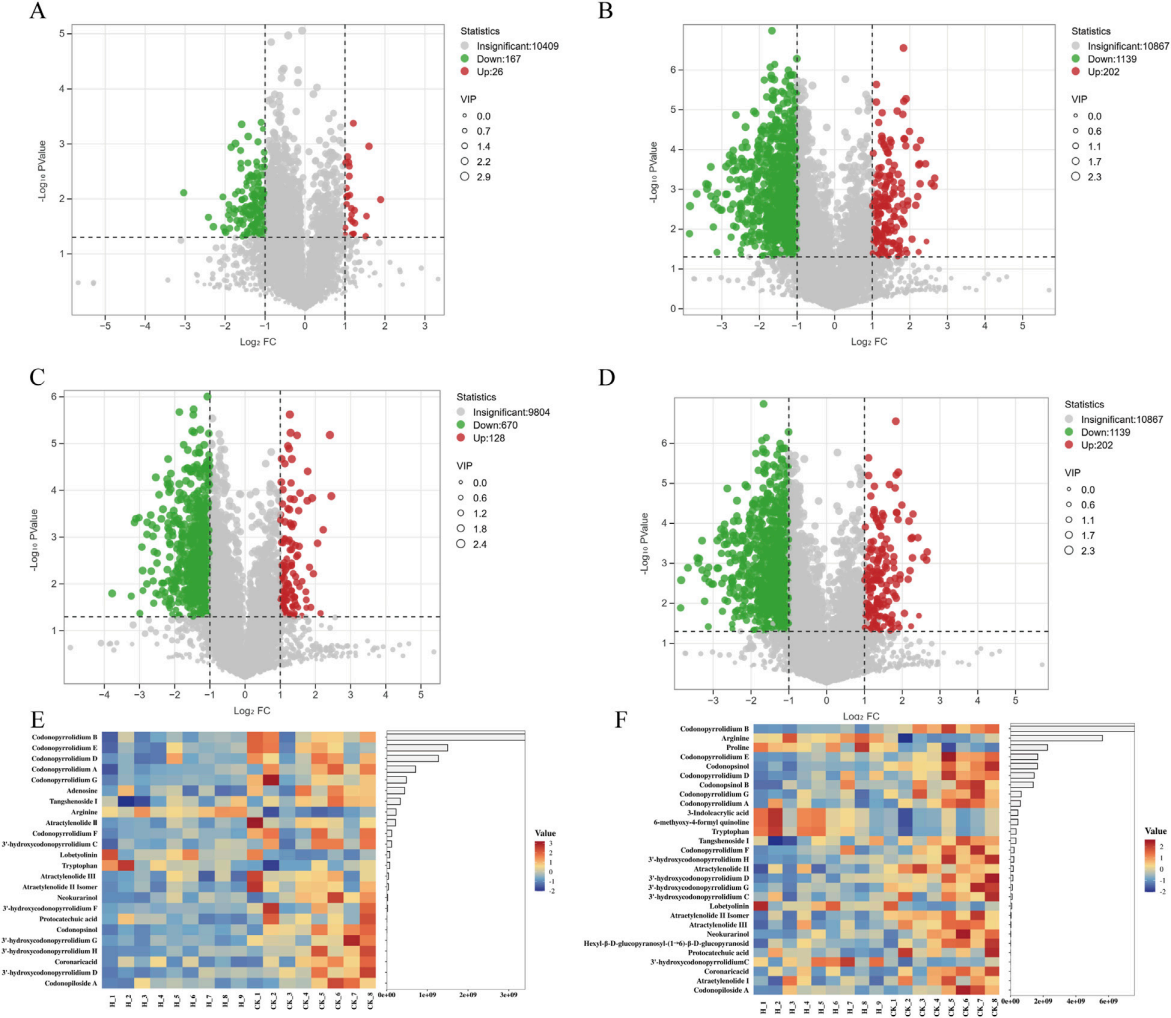
Volcano plot and heat map were used to illustrate the number of differential metabolites. **(A)** High vs. Control differential metabolite volcano plot in positive mode and **(B)** in negative mode for Pingshun; **(C)** High vs. Control differential metabolite volcano plot in positive mode and **(D)** in negative mode for Huguan; **(E)** heat map of differential metabolite expression between High and Control for Pingshun; **(F)** heat map of differential metabolite expression between High and Control for Huguan. note: Volcano plots was performed with selected metabolites based on VIP>1, |log2FC|> 1, and *P*-value < 0.05.

The distribution of metabolites was presented with Volcano plots and heatmaps (Figure 4). The volcano plot analysis revealed 193 differential metabolites with 167 reduced and 26 elevated in the ESI + mode, 1341 differential metabolites with 1139 reduced and 202 elevated in the ESI- mode for Pingshun (Figures 4A,B). The volcano plot analysis revealed 998 differential metabolites with 870 reduced and 128 elevated in the ESI + mode, 1341 differential metabolites with 1139 reduced and 202 elevated in the ESI- mode for Huguan (Figures 4C,D). Among these, the levels of 4 and 21 metabolites were significantly elevated and decreased tentatively identified, respectively, in high compared to control group for Pingshun (Figure 4E and detailed in Supplementary Table S8). Similarly, 7 metabolites were upregulated, and 22 were downregulated identified, respectively, in high compared to control group for Huguan (Figure 4F and detailed in Supplementary Table S9).

### 3.7 Effect of PGR on the chemical constituents of CR

**FIGURE 5:**
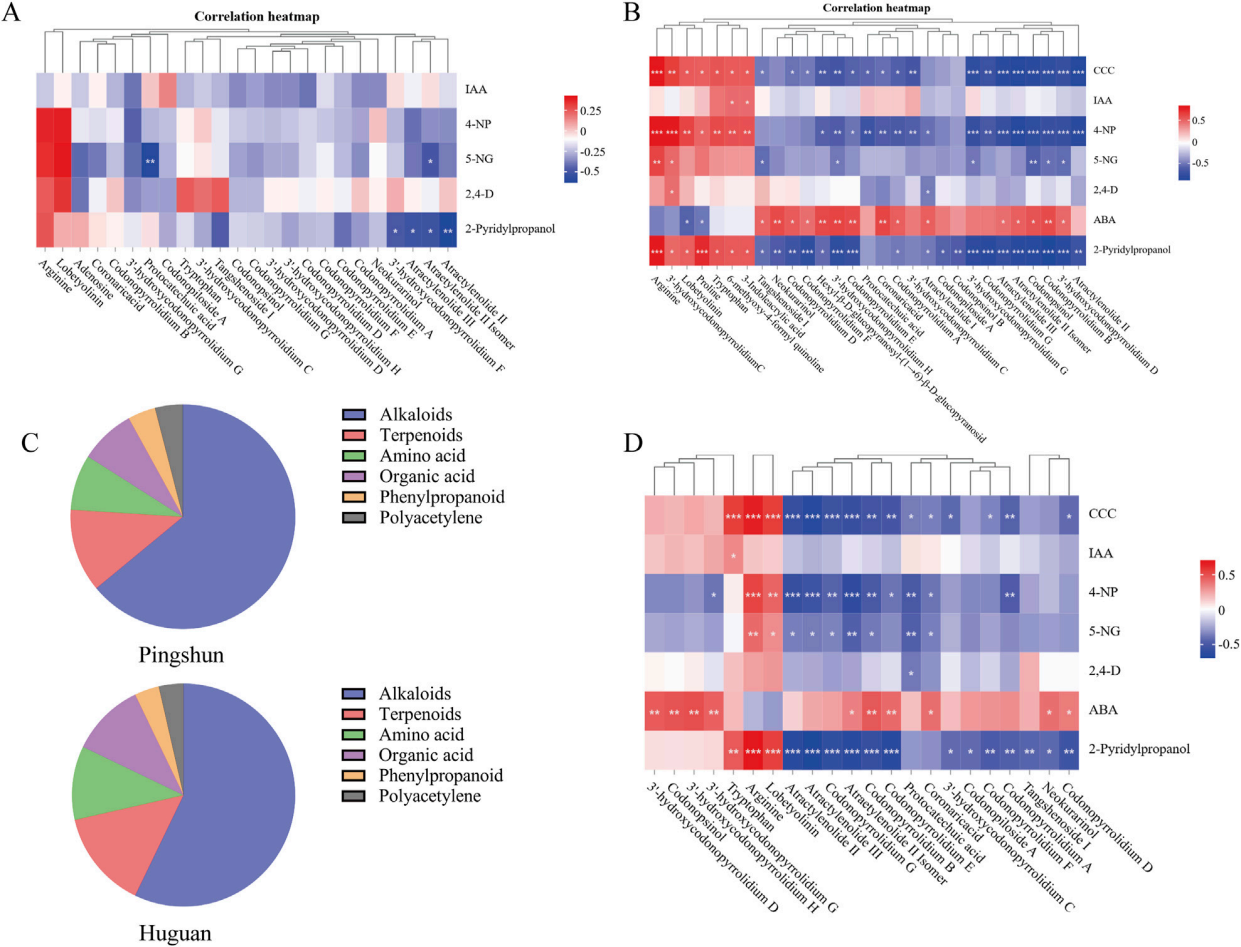
The relation analysis between PGRs and differential metabolites. **(A)** Clustering correlation heatmap for Pingshun; **(B)** Clustering correlation heatmap for Huguan; **(C)** Distribution of metabolite types; **(D)** Clustering correlation heatmap for all samples. Significance levels (*P*-values) are indicated in parentheses. The color bar denotes the strength of the correlation, with red representing positive correlations and blue indicating negative correlations. ^***^:*P* < 0.001, ^**^:*P* < 0.01, ^*^:*P* < 0.05.

In Pingshun CR, the concentration of certain components exhibited significant changes with varying levels of fertilizer application. Some significantly different alkaloids and terpenoids components decreased significantly with the increase in fertilization amount, such as atractylenolide III, atraetylenolide II isomer, atractylenolide Ⅱ, and 3′-hydroxycodonopyrrolidium F (Figure 5A). In contrast, the content of amino acid and polyacetylene components increased significantly, such as arginine, tryptophan, and lobetyolinin (Figure 5A).

In Huguan CR, the concentration of certain components exhibited significant changes with varying levels of fertilizer application. Specifically, the levels of compounds such as tangshenoside I, codonopiloside A, codonopyrrolidium B, etc., decreased notably as fertilizer amounts increased (Figure 5B). In contrast, the concentrations of amino acids, including arginine, tryptophan, and proline, showed a significant rise with higher fertilizer application (Figure 5B). Furthermore, compounds such as 3-indoleacrylic acid, a metabolite of tryptophan, 3′-hydroxycodonopyrrolidium C, lobetyolinin, and 6-methoxy-4-formyl quinoline also demonstrated a marked increase in concentration within the CR (Figure 5B). In addition, fertilizer ABA show the opposite correlation, the levels of alkaloids, organic acid, phenylpropanoid and terpenoids compounds increased notably as fertilizer amounts increased.

A comparative analysis of the alterations in metabolites between the Huguan and Pingshun CR reveals a notable increase in amino acid components post-fertilization, specifically arginine, tryptophan, and proline (Figure 5D). Conversely, there was a significant reduction in the levels of alkaloids, organic acids, phenylpropanoids, and terpenoid compounds, including codonopsinol, codonopyrrolidium B, astragaloside III, and astragaloside II (Figure 5D).

In distribution of metabolite types following fertilization, the main ingredients of differential metabolites are alkaloids in CR (Figure 5C). A specific type of agricultural fertilizer product incorporates multiple PGRs that collectively influence CR, either promoting or inhibiting the production of its secondary metabolites. For instance, the reduction in the levels of secondary metabolites such as codonopsinol and codonopyrrolidium B is primarily attributed to the inhibitory actions of CCC, 4-NP, and 2-pyridinepropanol, alongside the promoting effect of ABA. Recent studies have highlighted that CCC, 4-NP, and 5-NG exhibit toxicity and present significant health risks to humans (Kang et al., 2024; Wang et al., 2023).

## 4 Conclusion

This study has established a simple, effective, and high-throughput UPLC-MS/MS methodology for the multi-residue analysis of PGRs in CR, a widely utilized root and rhizome herb in traditional Chinese medicine. The chromatographic and spectrometric conditions were optimized to ensure the requisite separation, sensitivity, and specificity for analyzing 39 distinct PGRs. The established method was applied to analyze 102 batches of commercial product samples and 72 batches of field trial samples, in which the residue of 10 PGRs and 7 PGRs were detected, respectively. Furthermore, we used plant metabolomics to analyze metabolites changes in CR after fertilizer application, identifying shifts in secondary metabolites in the Huguan and Pingshun regions. A correlation analysis was conducted to examine the intrinsic relationship between PGRs and the secondary metabolites of CR. The findings indicated that applying PGRs enhances the synthesis of amino acid metabolites while concurrently inhibiting the biosynthesis of alkaloids. This study focuses on the quality and safety issues arising from the blind use of PGRs in the production of CR. This study provides a framework to support the rational and standardized cultivation and quality control necessary for the sustainable development of Traditional Chinese Medicine.

## Data Availability

The original contributions presented in the study are included in the article/Supplementary Material, further inquiries can be directed to the corresponding author.
