# Supplementary material for: A quantitative LC-MS/MS method for residues analysis of 62 plant growth regulators and the investigation of their effects on the quality of traditional Chinese medicinal: Codonopsis Radix as a case study
Source: Front Chem. 2025 Jul 15;13:1587915. doi: 10.3389/fchem.2025.1587915 (PMC12323384; doi:10.3389/fchem.2025.1587915)
Supplement: Supplementary file 1 [file DataSheet1.docx]

Supplementary Material

. **A quantitative LC-MS/MS method for analysis residues of 62 plant growth regulators and the investigation of their effects on the quality of traditional Chinese medicinal: *Codonopsis Radix* as a case study**

Fig. S1 Fertilization scheme of *C. pilosula*

Fig.S2 Extraction efficiency of 23 plant growth regulators with different extraction salts(n=3)

Fig.S3 Extraction efficiency of plant growth regulator with different proportion of extraction

Fig. S4 Recoveries of 32 PGRs in different purification methods(n=3)

Fig.S5 Permutation test plots (200 permutation tests) of PLS-DA/OPLS-DA model in positive and negative ion mode for Pingshun and Huguan (A) Permutation in positive mode in Pingshun of PLS-DA; (B) Permutation in negative mode in Pingshun of PLS-DA; (C) Permutation in positive mode in Huguan of PLS-DA; (D) Permutation in negative mode in Huguan of PLS-DA; (E) Permutation in positive mode in Pingshun of OPLS-DA; (F) Permutation in negative mode in Pingshun of OPLS-DA; (G) Permutation in positive mode in Huguan of OPLS-DA; (H) Permutation in negative mode in Huguan of OPLS-DA

Table S1 Sample information of CR with different fertilization treatments

Table S2 PH of 9 extraction schemes(n=3)

Table S3 Different extraction solvent ratios, extraction methods and times(n=3)

Table S4 Spiked recoveries and relative standard deviation (RSDs) of 62 plant growth regulators in CR (n=3)

Table S5 Sample information and PGRs residues detected of CR with102 batches

Table S6 Components identified in CR extract by UPLC-Q-Orbitrap MS

Table S7 LC-HR-MS/MS methodological investigation results

Table S8 The changes in the differential metabolites for Pingshun

Table S9 The changes in the differential metabolites for Huguan

## 1. Chemicals and reagents

Certified reference standards of PGRs, including 1,3-diphenylurea(DPU), 1-naphthaleneacetamide (1-NAD), 2-(3,4-dichlorophenoxy)-N,N-diethylethanamine (DCPTA), 2,4-dichlorophenoxyacetic acid (2,4-D), 2,4,5-trichlorophenoxyacetic acid (2,4,5-T), 2-naphthoxyacetic acid (2-NOA), 4-bromophenoxyacetic acid (4-BPA), 4-chlorphenoxyacetic acid (4-CPA), 4-fluorophenoxyacetic acid (4-FPA), 4-iodophenoxyacetic acid (4-IPA), sodium 4-nitrophenolate (4-NP), sodium 5-nitroguaiacolate (5-NG), 6-benzylaminopurine (6-BA), abscisic acid (ABA), ancymidol, atrazine (ATZ), chlormequat chloride (CCC), forchlorfenuron (CPPU), cyclanilide, ethylchlozate, gibberellic acid 3 (GA3), gibberellic acid 7 (GA7), inabenfide, indole-3-acetic acid (IAA), indole-3-butyric acid (IBA), indole-3-propionic acid (IPA), kinetin (KT), mepiquat chloride (Pix), N6-(2-isopentynyl)-adenine (2iP), paclobutrazol (PBZ), prohexadione, prohydrojasmon (PHJ), simazine (SMZ), thidiazuron (TDZ), tebuconazole (TBZ), thiabendazole (ThiBZ), triapenthenol(TPN), trinexapac ethyl (TE), uniconazole (UCZ), and the internal standard, Atrazine-d5 (ATZ-*d5*), were purchased from A Chemtek Inc. (Woburn, MA, USA). 2-Pyridylpropanol, benzanilide, brassinolide (BR), carbaryl, chlorphonium chloride, cycloheximide, daminozide, dichlorprop, flumetralin, flurprimidol, and gibberellic acid 4 (GA4) were purchased from Alta Scientific Co., Ltd. (Tianjin, China). 2,3,5-triiodobenzoic acid (TIBA), butralin, chlorpropham, cloprop, diethyl aminoethyl hexanoate (DA-6), dikegulac monohydrate, diniconazole, ethy 1-naphthaleneacetate, mefluidide, pyraflufen-ethyl, pyribenzoxim, and tribufos were purchased from Dr. Ehrenstorfer GmbH (Augsburg; Germany). Commercial PGR product “Dangshenqifei” (including 4-NP, CCC and 2-Pyridylpropanol) were purchased from Yishun AIB Company (Gansu, China). Arginine, phenylalanine, proline, adenosine, tryptophan, and lobetyolin were purchased from National Institutes for Food and Drug Control (NIFDC, Beijing, China).

**2. Optimization of sample preparation methodology**

**2.1 Extraction salt and pH**

The QuEChERS method, recognized as a preferred technique for sample preparation in pesticide residue detection, is noted for its operational simplicity. Nevertheless, the recovery rates of pesticides exhibit considerable variability depending on the extraction salts employed. This study devised nine extraction schemes to examine eight different types of extraction salts and to assess the extraction pH, show in **Table S2**.

**2.2 Extraction solvent, technique and time**

Using the extraction Scheme C with the addition of salt, the mean recovery rate (n=3) was employed as a metric to examine the effects of varying acetonitrile-to-water ratios, extraction techniques, and extraction durations, as detailed in **Table S3**.

**2.3 SPE cleanup**

In the QuEChERS method, common dispersive solid-phase extraction materials include PSA, silica gel, C18, and GCB. PSA and silica gel target strongly polar impurities like sugars and fatty acids. C18 removes non-polar impurities such as sterols and alkenes. GCB is effective for planar compounds, eliminating carotenoids, chlorophyll, and sterols. Furthermore, the purification process can variably impact the recovery rate of target compounds similar to impurities, leading some methods to skip purification.

*
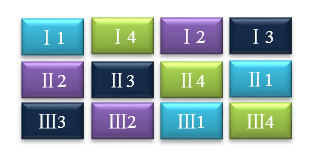
*

Fig. S1 Fertilization scheme of *C. pilosula*

**
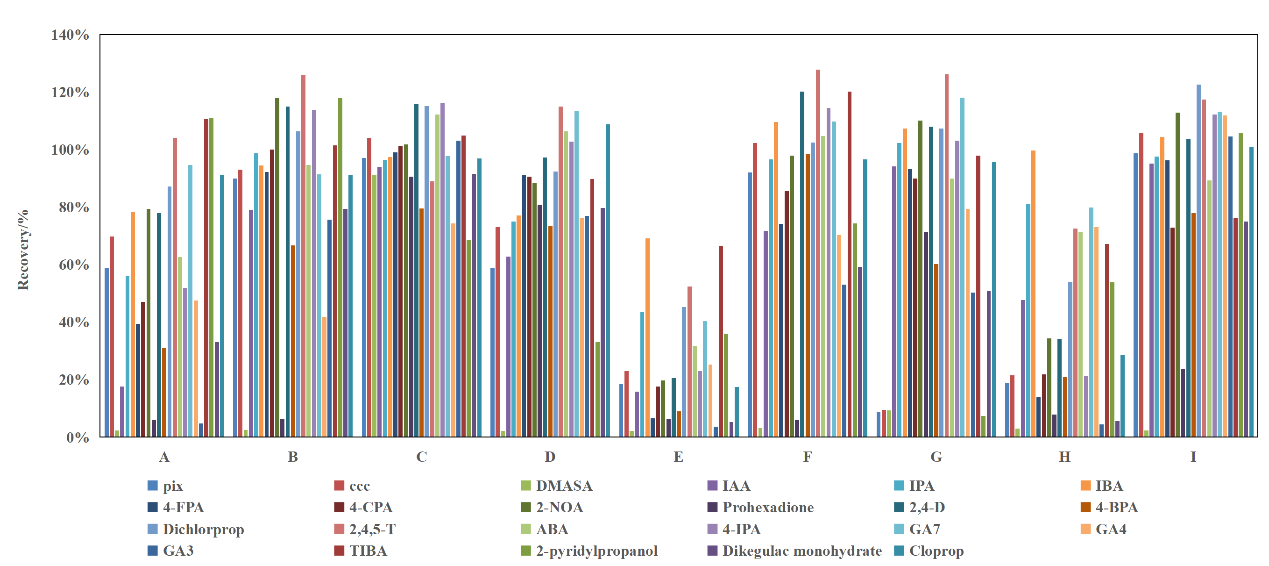
**

Fig.S2 Extraction efficiency of 23 plant growth regulators with different extraction salts(n=3)


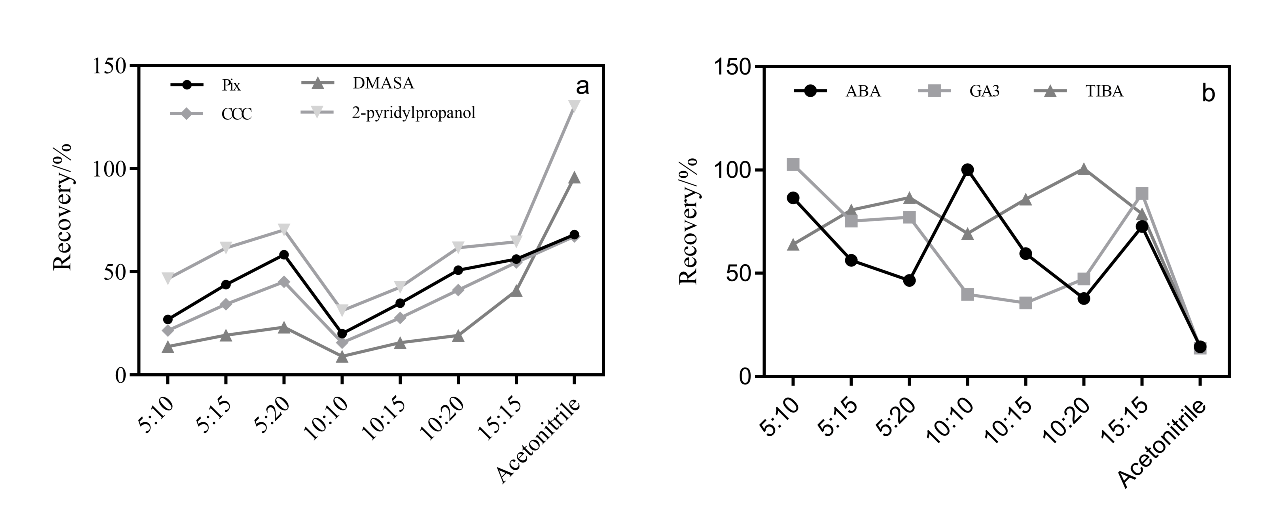


Fig.S3 Extraction efficiency of plant growth regulator with different proportion of extraction solvent(n=3)

**
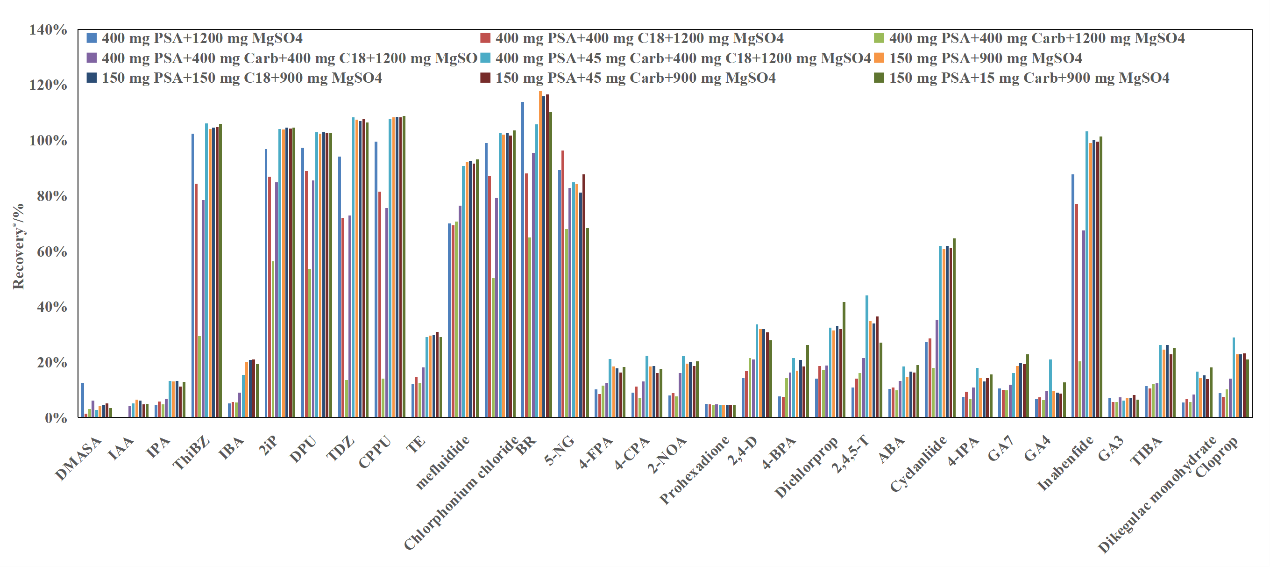
**

Fig. S4 Recoveries of 32 PGRs in different purification methods(n=3)


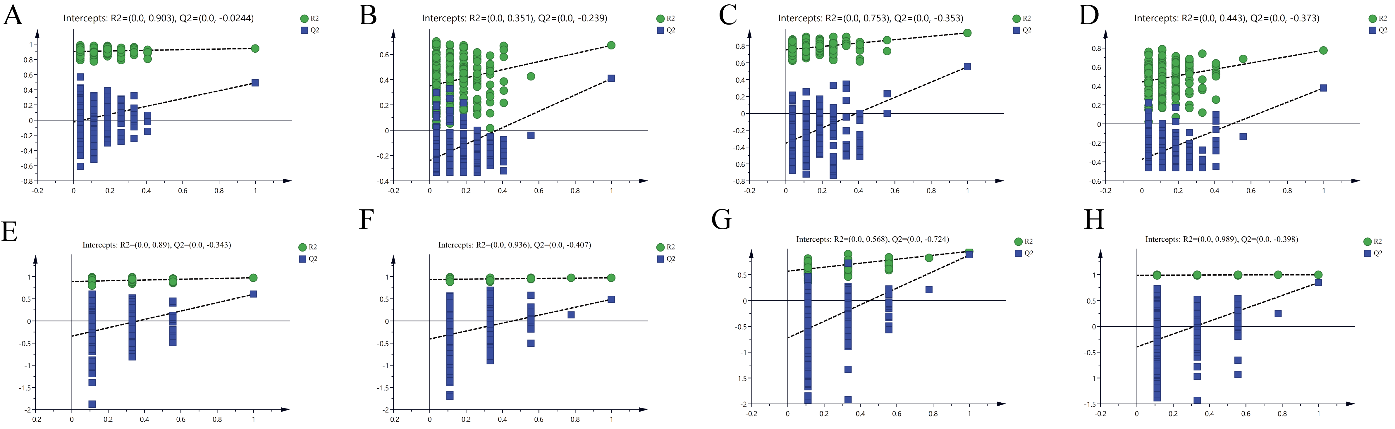


Fig.S5 Permutation test plots (200 permutation tests) of PLS-DA/OPLS-DA model in positive and negative ion mode for Pingshun and Huguan (A) Permutation in positive mode in Pingshun of PLS-DA; (B) Permutation in negative mode in Pingshun of PLS-DA; (C) Permutation in positive mode in Huguan of PLS-DA; (D) Permutation in negative mode in Huguan of PLS-DA; (E) Permutation in positive mode in Pingshun of OPLS-DA; (F) Permutation in negative mode in Pingshun of OPLS-DA; (G) Permutation in positive mode in Huguan of OPLS-DA; (H) Permutation in negative mode in Huguan of OPLS-DA

Table S1 Sample information of CR with different fertilization treatments

| NO. | Batches | Habitats | Fertilizer application times | Type |
| --- | --- | --- | --- | --- |
| 1 | HG01 | Huguan | 0 | Control_1 |
| 2 | HG02 | Huguan | 0 | Control_2 |
| 3 | HG03 | Huguan | 0 | Control_3 |
| 4 | HG04 | Huguan | 0 | Control_4 |
| 5 | HG05 | Huguan | 0 | Control_5 |
| 6 | HG06 | Huguan | 0 | Control_6 |
| 7 | HG07 | Huguan | 0 | Control_7 |
| 8 | HG08 | Huguan | 0 | Control_8 |
| 9 | HG09 | Huguan | 0 | Control_9 |
| 10 | HG10 | Huguan | 1 | Low_1 |
| 11 | HG11 | Huguan | 1 | Low_2 |
| 12 | HG12 | Huguan | 1 | Low_3 |
| 13 | HG13 | Huguan | 1 | Low_4 |
| 14 | HG14 | Huguan | 1 | Low_5 |
| 15 | HG15 | Huguan | 1 | Low_6 |
| 16 | HG16 | Huguan | 1 | Low_7 |
| 17 | HG17 | Huguan | 1 | Low_8 |
| 18 | HG18 | Huguan | 1 | Low_9 |
| 19 | HG19 | Huguan | 2 | Middle_1 |
| 20 | HG20 | Huguan | 2 | Middle_2 |
| 21 | HG21 | Huguan | 2 | Middle_3 |
| 22 | HG22 | Huguan | 2 | Middle_4 |
| 23 | HG23 | Huguan | 2 | Middle_5 |
| 24 | HG24 | Huguan | 2 | Middle_6 |
| 25 | HG25 | Huguan | 2 | Middle_7 |
| 26 | HG26 | Huguan | 2 | Middle_8 |
| 27 | HG27 | Huguan | 2 | Middle_9 |
| 28 | HG28 | Huguan | 3 | High_1 |
| 29 | HG29 | Huguan | 3 | High_2 |
| 30 | HG30 | Huguan | 3 | High_3 |
| 31 | HG31 | Huguan | 3 | High_4 |
| 32 | HG32 | Huguan | 3 | High_5 |
| 33 | HG33 | Huguan | 3 | High_6 |
| 34 | HG34 | Huguan | 3 | High_7 |
| 35 | HG35 | Huguan | 3 | High_8 |
| 36 | HG36 | Huguan | 3 | High_9 |
| 37 | PS01 | Pinshun | 0 | Control_1 |
| 38 | PS02 | Pinshun | 0 | Control_2 |
| 39 | PS03 | Pinshun | 0 | Control_3 |
| 40 | PS04 | Pinshun | 0 | Control_4 |
| 41 | PS05 | Pinshun | 0 | Control_5 |
| 42 | PS06 | Pinshun | 0 | Control_6 |
| 43 | PS07 | Pinshun | 0 | Control_7 |
| 44 | PS08 | Pinshun | 0 | Control_8 |
| 45 | PS09 | Pinshun | 0 | Control_9 |
| 46 | PS10 | Pinshun | 1 | Low_1 |
| 47 | PS11 | Pinshun | 1 | Low_2 |
| 48 | PS12 | Pinshun | 1 | Low_3 |
| 49 | PS13 | Pinshun | 1 | Low_4 |
| 50 | PS14 | Pinshun | 1 | Low_5 |
| 51 | PS15 | Pinshun | 1 | Low_6 |
| 52 | PS16 | Pinshun | 1 | Low_7 |
| 53 | PS17 | Pinshun | 1 | Low_8 |
| 54 | PS18 | Pinshun | 1 | Low_9 |
| 55 | PS19 | Pinshun | 2 | Middle_1 |
| 56 | PS20 | Pinshun | 2 | Middle_2 |
| 57 | PS21 | Pinshun | 2 | Middle_3 |
| 58 | PS22 | Pinshun | 2 | Middle_4 |
| 59 | PS23 | Pinshun | 2 | Middle_5 |
| 60 | PS24 | Pinshun | 2 | Middle_6 |
| 61 | PS25 | Pinshun | 2 | Middle_7 |
| 62 | PS26 | Pinshun | 2 | Middle_8 |
| 63 | PS27 | Pinshun | 2 | Middle_9 |
| 64 | PS28 | Pinshun | 3 | High_1 |
| 65 | PS29 | Pinshun | 3 | High_2 |
| 66 | PS30 | Pinshun | 3 | High_3 |
| 67 | PS31 | Pinshun | 3 | High_4 |
| 68 | PS32 | Pinshun | 3 | High_5 |
| 69 | PS33 | Pinshun | 3 | High_6 |
| 70 | PS34 | Pinshun | 3 | High_7 |
| 71 | PS35 | Pinshun | 3 | High_8 |
| 72 | PS36 | Pinshun | 3 | High_9 |

Table S2 PH of 9 extraction schemes(n=3)

| Name | Salting-out extraction | Extraction solvent | ACN layer pH | H_2_O layer pH |
| --- | --- | --- | --- | --- |
| A | 6 g MgSO_4_, 1.5 g NaCl | ACN | 5.64 | 7.60 |
| B | 6 g MgSO_4_, 1.5 g NaOAc | 1% acetic acid in ACN | 4.26 | 5.70 |
| C | 4 g MgSO_4_, 1 g NaCl, 1 g Na_3_Cit., 0.5 g Na_2_HCit | ACN | 4.06 | 3.78 |
| D | 6 g MgSO_4_, 1.5 g NaCl | 1% acetic acid in ACN | 3.30 | 3.12 |
| E | 7.5 g NH_4_OAc | 1% acetic acid in ACN | 5.10 | 7.13 |
| F | 6 g MgSO_4_, 1.5 g NH_4_OAc | 1% acetic acid in ACN | 4.39 | 5.72 |
| G | 7.5 g NH_4_Cl | ACN | 4.97 | 4.33 |
| H | 7.5 g, NH_4_O_2_CH | 1% acetic acid in ACN | 3.94 | 6.10 |
| I | 6 g MgSO_4_, 1.5 g NH_4_O_2_CH | 1% acetic acid in ACN | 3.18 | 4.67 |

Table S3 Different extraction solvent ratios, extraction methods and times(n=3)

| Name | The volume of H_2_O | The volume of ACN | Ratios | Extraction method | Time (min) |
| --- | --- | --- | --- | --- | --- |
| C1 | 5 | 10 | 2:1 | Shaken | 5 |
| C2 | 5 | 15 | 3:1 | Shaken | 5 |
| C3 | 5 | 20 | 4:1 | Shaken | 5 |
| C4 | 10 | 10 | 1:1 | Shaken | 5 |
| C5 | 10 | 15 | 3:2 | Shaken | 5 |
| C6 | 10 | 20 | 2:1 | Shaken | 5 |
| C7 | 15 | 15 | 1:1 | Shaken | 5 |
| C8 | - | 20 | - | Shaken | 5 |
| C9 | 15 | 15 | 1:1 | Ultrasound | 5 |
| C10 | 15 | 15 | 1:1 | Shaken | 10 |
| C11 | 15 | 15 | 1:1 | Shaken | 30 |

Table S4 Spiked recoveries and relative standard deviation (RSDs) of 62 plant growth regulators in CR (n=3)

| PGRs | Spiked (μg/kg) | Recovery (%) | | RSD (%) | |
| --- | --- | --- | --- | --- | --- |
|  |  | Intra-day | Inter-day | Intra-day | Inter-day |
| CCC | 4.90 | 115.67 | 92.95 | 18.44 | 16.63 |
|  | 9.80 | 123.65 | 106.80 | 6.37 | 17.46 |
|  | 49.00 | 96.68 | 88.84 | 7.50 | 10.27 |
| PIX | 24.75 | 122.94 | 122.53 | 11.35 | 15.87 |
|  | 49.50 | 99.68 | 100.92 | 10.16 | 7.56 |
|  | 198.00 | 91.97 | 92.57 | 5.87 | 3.96 |
| 2-Pyridylpropanol | 24.53 | 93.37 | 99.50 | 15.30 | 18.52 |
|  | 49.05 | 107.11 | 114.14 | 19.26 | 17.61 |
|  | 490.50 | 128.17 | 129.47 | 4.50 | 8.88 |
| DMASA | 99.50 | 69.99 | 75.41 | 18.55 | 18.82 |
|  | 199.00 | 73.15 | 73.3 | 5.62 | 16.28 |
|  | 497.50 | 108.8 | 98.58 | 5.57 | 13.64 |
| KT | 2.48 | 107.22 | 97.02 | 19.72 | 17.98 |
|  | 9.90 | 97.56 | 98.28 | 5.37 | 6.54 |
|  | 49.50 | 97.96 | 100.91 | 4.77 | 3.99 |
| ThiBZ | 0.50 | 121.48 | 129.04 | 16.53 | 14.14 |
|  | 9.99 | 95.75 | 98.93 | 7.09 | 13.24 |
|  | 49.95 | 105.95 | 102.45 | 2.05 | 5.03 |
| 2iP | 9.85 | 99.73 | 99.36 | 10.24 | 9.80 |
|  | 49.25 | 99.14 | 100.87 | 5.15 | 3.79 |
|  | 197.00 | 100.91 | 101.55 | 1.83 | 2.32 |
| GA3 | 98.00 | 77.30 | 83.23 | 15.64 | 19.5 |
|  | 196.00 | 75.16 | 76.95 | 16.26 | 15.27 |
|  | 490.00 | 103.83 | 94.72 | 12.35 | 19.66 |
| 6-BA | 2.50 | 114.58 | 122.77 | 16.67 | 14.9 |
|  | 9.99 | 87.11 | 98.26 | 5.09 | 13.92 |
|  | 49.95 | 120.65 | 117.04 | 11.37 | 7.98 |
| Dikegulac monohydrate | 4.97 | 92.22 | 69.05 | 19.31 | 19.32 |
|  | 9.94 | 97.22 | 88.13 | 7.31 | 19.77 |
|  | 49.70 | 94.71 | 89.10 | 17.19 | 13.17 |
| 4-FPA | 49.00 | 82.49 | 69.82 | 15.77 | 14.21 |
|  | 196.00 | 82.12 | 87.71 | 17.09 | 19.88 |
|  | 490.00 | 127.37 | 121.78 | 5.14 | 16.06 |
| Prohexadione | 197.80 | 107.82 | 85.54 | 14.29 | 19.44 |
|  | 791.20 | 81.61 | 80.77 | 16.52 | 15.62 |
|  | 197.80 | 99.39 | 91.73 | 11.03 | 16.43 |
| IAA | 196.00 | 107.58 | 98.19 | 15.56 | 18.97 |
|  | 490.00 | 93.77 | 88.52 | 10.29 | 17.74 |
|  | 980.00 | 91.79 | 99.12 | 5.99 | 13.52 |
| DA-6 | 0.49 | 80.02 | 89.92 | 18.93 | 19.62 |
|  | 9.89 | 101.74 | 96.88 | 4.48 | 4.81 |
|  | 49.45 | 99.20 | 98.56 | 5.37 | 4.43 |
| 4-NP | 4.90 | 128.22 | 123.69 | 11.04 | 19.15 |
|  | 24.50 | 74.33 | 91.33 | 0.03 | 19.39 |
|  | 49.00 | 118.92 | 103.18 | 4.78 | 17.45 |
| 5-NG | 98.00 | 94.9 | 79.06 | 13.43 | 18.00 |
|  | 196.00 | 90.00 | 89.38 | 15.47 | 18.87 |
|  | 490.00 | 117.80 | 102.94 | 7.40 | 16.69 |
| 1-NAD | 4.95 | 85.40 | 101.66 | 3.34 | 16.75 |
|  | 9.90 | 97.21 | 107.44 | 7.25 | 19.99 |
|  | 49.50 | 98.41 | 101.89 | 1.45 | 6.51 |
| ABA | 98.50 | 123.38 | 120.19 | 15.00 | 18.65 |
|  | 197.00 | 92.68 | 103.86 | 15.42 | 17.13 |
|  | 492.50 | 89.47 | 102.89 | 15.47 | 15.63 |
| Actidione | 4.91 | 123.20 | 119.97 | 15.69 | 13.84 |
|  | 9.82 | 115.73 | 112.72 | 11.57 | 7.86 |
|  | 49.10 | 103.09 | 101.02 | 2.28 | 4.06 |
| 4-CPA | 49.00 | 110.49 | 110.55 | 18.29 | 17.43 |
|  | 196.00 | 105.99 | 101.99 | 7.21 | 18.09 |
|  | 490.00 | 96.37 | 97.78 | 13.83 | 9.21 |
| IPA | 9.95 | 62.76 | 62.46 | 15.45 | 19.42 |
|  | 49.75 | 100.06 | 101.36 | 11.22 | 10.74 |
|  | 199.00 | 100.95 | 101.79 | 8.83 | 5.92 |
| TDZ | 1.00 | 68.53 | 75.75 | 7.56 | 16.26 |
|  | 9.99 | 104.94 | 106.31 | 12.77 | 10.44 |
|  | 49.95 | 99.04 | 101.97 | 6.45 | 7.21 |
| DCPTA | 1.00 | 94.40 | 97.36 | 17.83 | 18.37 |
|  | 9.99 | 90.98 | 92.76 | 6.97 | 18.75 |
|  | 49.95 | 99.6 | 103.25 | 14.2 | 9.28 |
| SMZ | 9.95 | 83.00 | 84.78 | 9.73 | 16.07 |
|  | 49.75 | 96.11 | 94.61 | 4.50 | 3.65 |
|  | 199.00 | 99.54 | 100.87 | 1.02 | 3.70 |
| Ancymidol | 4.91 | 123.20 | 119.97 | 15.69 | 13.84 |
|  | 9.82 | 115.73 | 112.72 | 11.57 | 7.86 |
|  | 49.10 | 103.09 | 101.02 | 2.28 | 4.06 |
| 4-BPA | 94.30 | 86.21 | 86.53 | 14.55 | 19.11 |
|  | 188.60 | 112.62 | 97.27 | 6.39 | 18.27 |
|  | 471.50 | 99.17 | 104.43 | 14.28 | 15.13 |
| IBA | 98.00 | 81.21 | 106.6 | 19.45 | 15.57 |
|  | 490.00 | 100.47 | 97.31 | 5.32 | 9.11 |
|  | 980.00 | 92.22 | 95.29 | 5.98 | 6.15 |
| 4-IPA | 99.20 | 70.05 | 72.69 | 16.41 | 19.98 |
|  | 198.40 | 99.93 | 102.35 | 14.28 | 16.34 |
|  | 496.00 | 96.55 | 101.43 | 17.94 | 17.13 |
| Cloprop | 24.77 | 102.11 | 101.30 | 17.46 | 18.91 |
|  | 49.53 | 106.17 | 98.42 | 3.95 | 16.26 |
|  | 198.12 | 94.79 | 92.83 | 14.01 | 17.75 |
| 2-NOA | 24.95 | 97.96 | 94.95 | 19.12 | 16.91 |
|  | 49.9 | 106.66 | 89.31 | 11.20 | 18.66 |
|  | 199.6 | 90.74 | 89.28 | 6.13 | 16.4 |
| Mefluidide | 2.44 | 85.24 | 83.18 | 15.84 | 8.03 |
|  | 9.75 | 104.62 | 107.17 | 5.98 | 7.08 |
|  | 48.75 | 97.57 | 97.92 | 3.28 | 3.56 |
| 2,4-D | 49.00 | 86.32 | 80.08 | 18.32 | 16.97 |
|  | 196.00 | 87.8 | 81.7 | 5.98 | 17.41 |
|  | 490.00 | 98.93 | 97.62 | 2.35 | 9.09 |
| SOK | 9.95 | 82.30 | 89.88 | 8.83 | 15.07 |
|  | 49.75 | 93.15 | 96.81 | 5.55 | 4.65 |
|  | 199.00 | 96.64 | 101.67 | 2.02 | 6.70 |
| ATZ | 0.50 | 91.43 | 102.88 | 5.47 | 19.42 |
|  | 9.90 | 100.71 | 96.88 | 4.48 | 4.81 |
|  | 49.50 | 101.11 | 105.28 | 9.44 | 6.32 |
| CPPU | 1.00 | 107.88 | 101.14 | 14.94 | 18.38 |
|  | 9.95 | 120.89 | 116.43 | 18.58 | 14.10 |
|  | 49.75 | 103.82 | 103.94 | 6.88 | 4.83 |
| GA7 | 23.75 | 98.13 | 87.51 | 17.17 | 17.96 |
|  | 47.5 | 71.08 | 85.76 | 12.93 | 19.35 |
|  | 190 | 89.33 | 82.91 | 11.56 | 18.25 |
| Benzanilide | 0.50 | 116.13 | 126.68 | 15.64 | 17.79 |
|  | 9.99 | 91.60 | 95.61 | 7.96 | 7.94 |
|  | 49.95 | 99.54 | 99.38 | 2.87 | 2.16 |
| GA4 | 93.60 | 83.96 | 84.87 | 6.59 | 5.51 |
|  | 187.20 | 79.2 | 79.22 | 14.02 | 12.26 |
|  | 468.00 | 83.00 | 80.61 | 17.64 | 19.45 |
| DPU | 2.48 | 86.49 | 89.96 | 14.18 | 11.84 |
|  | 9.92 | 96.57 | 102.71 | 3.65 | 5.89 |
|  | 49.60 | 101.24 | 98.73 | 5.22 | 3.97 |
| Ethychlozate | 49.00 | 105.66 | 110.79 | 19.7 | 16.26 |
|  | 196.00 | 87.57 | 100.59 | 16.09 | 17.45 |
|  | 490.00 | 111.63 | 109.42 | 8.59 | 15.94 |
| 2,4,5-T | 49.50 | 84.45 | 69.60 | 18.71 | 19.09 |
|  | 198.00 | 106.37 | 99.62 | 11.87 | 12.53 |
|  | 495.00 | 106.47 | 103.56 | 15.26 | 9.52 |
| Dichlorprop | 49.85 | 82.00 | 81.49 | 8.75 | 18.74 |
|  | 199.40 | 80.99 | 90.67 | 10.51 | 13.75 |
|  | 498.50 | 102.83 | 98.62 | 11.99 | 9.87 |
| TE | 9.92 | 65.12 | 70.49 | 17.82 | 18.87 |
|  | 49.60 | 104.97 | 100.83 | 11.06 | 11.95 |
|  | 198.40 | 104.82 | 106.52 | 1.31 | 3.48 |
| TIBA | 198.00 | 76.02 | 100.1 | 15.49 | 19.43 |
|  | 495.00 | 88.90 | 99.82 | 19.18 | 18.34 |
|  | 990.00 | 100.78 | 107.08 | 3.90 | 12.87 |
| Inabenfide | 4.90 | 91.75 | 89.94 | 11.00 | 18.02 |
|  | 9.80 | 71.33 | 65.00 | 15.78 | 19.83 |
|  | 49.00 | 94.89 | 100.08 | 11.80 | 19.74 |
| Cyclanilide | 9.90 | 89.82 | 69.95 | 15.38 | 19.75 |
|  | 49.50 | 82.69 | 86.37 | 15.76 | 17.9 |
|  | 198.00 | 96.84 | 100.30 | 5.73 | 7.08 |
| Flurprimidol | 4.98 | 91.83 | 85.43 | 16.68 | 12.50 |
|  | 9.96 | 92.28 | 87.70 | 15.23 | 15.32 |
|  | 49.80 | 99.57 | 101.82 | 0.61 | 6.32 |
| BR | 49.32 | 111.36 | 108.51 | 15.49 | 17.35 |
|  | 197.28 | 106.94 | 104.61 | 3.15 | 6.74 |
|  | 493.20 | 92.03 | 96.49 | 3.91 | 6.83 |
| PHJ | 82.50 | 100.95 | 102.78 | 5.50 | 7.31 |
|  | 165.00 | 98.80 | 103.97 | 5.86 | 6.16 |
|  | 412.50 | 100.73 | 106.29 | 1.64 | 5.25 |
| PBZ | 4.99 | 94.24 | 95.91 | 9.98 | 5.93 |
|  | 9.98 | 85.15 | 99.29 | 9.84 | 15.73 |
|  | 49.90 | 107.81 | 107.05 | 4.79 | 3.96 |
| UCZ | 2.47 | 116.15 | 113.18 | 7.93 | 7.86 |
|  | 9.89 | 102.16 | 102.78 | 2.09 | 4.15 |
|  | 49.45 | 98.46 | 97.14 | 2.69 | 2.07 |
| TPN | 0.99 | 80.83 | 79.5 | 10.35 | 18.12 |
|  | 9.90 | 95.33 | 102.67 | 2.36 | 6.46 |
|  | 49.50 | 101.62 | 101.7 | 2.62 | 2.17 |
| CIPC | 49.85 | 115.64 | 119.23 | 15.43 | 18.49 |
|  | 199.40 | 98.2 | 103.23 | 5.06 | 10.51 |
|  | 498.50 | 98.73 | 106.41 | 15.91 | 13.29 |
| TBZ | 4.97 | 97.06 | 99.63 | 10.52 | 7.14 |
|  | 9.93 | 99.24 | 101.54 | 4.9 | 5.13 |
|  | 49.65 | 103.34 | 101.82 | 3.49 | 3.25 |
| Chlorphonium chloride | 2.50 | 90.90 | 90.02 | 8.52 | 9.53 |
|  | 9.99 | 93.56 | 105.95 | 5.05 | 13.94 |
|  | 49.95 | 100.75 | 99.10 | 2.62 | 2.39 |
| Ethy 1-naphthaleneacetate | 9.23 | 87.38 | 88.81 | 9.12 | 8.07 |
|  | 46.15 | 97.05 | 95.75 | 4.78 | 3.46 |
|  | 184.60 | 100.71 | 100.57 | 2.87 | 2.22 |
| Diniconazole | 2.47 | 112.88 | 104.00 | 19.86 | 14.03 |
|  | 9.90 | 102.38 | 105.80 | 6.70 | 8.18 |
|  | 49.48 | 102.24 | 98.66 | 3.21 | 4.57 |
| Pyraflufen-ethyl | 2.49 | 95.01 | 95.53 | 14.1 | 15.73 |
|  | 9.96 | 95.93 | 105.52 | 1.60 | 8.35 |
|  | 49.78 | 104.75 | 103.55 | 2.99 | 1.96 |
| Pyribenzoxim | 197.22 | 103.84 | 106.56 | 1.45 | 3.74 |
|  | 493.05 | 115.57 | 114.8 | 1.98 | 1.65 |
|  | 986.10 | 101.71 | 103.46 | 1.77 | 2.73 |
| Flumetralin | 99.90 | 90.29 | 94.53 | 14.19 | 12.96 |
|  | 199.80 | 103.47 | 109.05 | 5.01 | 6.79 |
|  | 499.50 | 100.97 | 100.91 | 3.13 | 4.10 |
| Butralin | 2.49 | 82.83 | 90.68 | 19.53 | 19.93 |
|  | 9.97 | 101.66 | 108.00 | 3.31 | 7.79 |
|  | 49.86 | 99.19 | 98.08 | 1.90 | 5.37 |
| DEF | 24.53 | 103.25 | 92.15 | 16.40 | 18.62 |
|  | 49.05 | 100.11 | 104.24 | 18.36 | 18.63 |
|  | 490.50 | 118.25 | 123.32 | 5.50 | 9.38 |

Table S5 Sample information and PGRs residues detected of CR with102 batches

| NO. | Habitats | Fertilizer application | CCC | IAA | 4-NP | Pix | UCZ | PBZ | 2-pyridylpropanol | Actidione | Indole-3-butyric acid | ABA |
| --- | --- | --- | --- | --- | --- | --- | --- | --- | --- | --- | --- | --- |
| 1 | Yunyang, Chongqing | - |  |  |  |  |  |  | 192.16 |  |  |  |
| 2 | Yunyang, Chongqing | - |  |  |  |  |  |  | 125.55 |  |  |  |
| 3 | Yunyang, Chongqing | - |  |  | 107.84 |  |  |  | 137.73 |  |  |  |
| 4 | Longnan, Gansu | - |  |  |  |  |  |  |  |  |  |  |
| 5 | Dingxi, Gansu | - | 4.05 |  | 118.47 |  |  |  | 152.63 |  |  |  |
| 6 | Shanxi | - |  | 382.71 | 220.87 |  |  |  |  |  |  |  |
| 7 | Yunyang, Chongqing | Dangshenqifei |  |  |  |  |  |  | 125.69 |  |  |  |
| 8 | Yunyang, Chongqing | Dangshenqifei |  | 470.95 | 272.48 |  |  |  | 85.97 |  |  |  |
| 9 | Yunyang, Chongqing | Dangshenqifei |  |  | 77.55 |  |  |  | 117.22 |  |  |  |
| 10 | Lanzhou, Gansu | Dangshenqifei | 11.74 |  | 30.70 |  |  |  | 158.07 |  |  |  |
| 11 | Lanzhou, Gansu | Dangshenqifei | 36.87 |  | 221.47 |  |  |  |  |  |  |  |
| 12 | Lanzhou, Gansu | Dangshenqifei | 76.40 |  |  |  |  |  | 324.50 |  |  |  |
| 13 | Lanzhou, Gansu | 5 times dose Dangshenqifei | 84.89 |  |  |  |  |  | 173.01 |  |  |  |
| 14 | Lanzhou, Gansu | 5 times dose Dangshenqifei | 179.91 |  | 22.26 |  |  |  | 234.55 |  |  |  |
| 15 | Lanzhou, Gansu | 5 times dose Dangshenqifei | 251.21 | 544.49 |  |  |  |  | 563.91 |  |  |  |
| 16 | Lanzhou, Gansu | Genyuan | 30.06 |  | 111.05 |  |  |  | 39.43 |  |  |  |
| 17 | Lanzhou, Gansu | Genyuan |  |  |  |  |  |  | 164.50 |  |  |  |
| 18 | Lanzhou, Gansu | Genyuan |  |  | 84.04 | 26.42 |  |  | 345.82 |  |  |  |
| 19 | Lanzhou, Gansu | 5 times dose Genyuan | 16.61 | 382.20 | 66.51 |  |  |  | 180.60 |  |  |  |
| 20 | Lanzhou, Gansu | 5 times dose Genyuan | 5.02 |  | 16.46 |  |  |  | 344.51 |  |  |  |
| 21 | Lanzhou, Gansu | 5 times dose Genyuan |  |  | 53.59 |  |  |  | 580.81 |  |  |  |
| 22 | Lanzhou, Gansu | Longgensheng |  |  | 25.49 |  |  |  | 108.72 |  |  |  |
| 23 | Lanzhou, Gansu | Longgensheng | 16.82 |  | 17.05 |  |  |  | 226.00 |  |  |  |
| 24 | Lanzhou, Gansu | Longgensheng | 7.70 |  |  |  |  |  | 404.28 |  |  |  |
| 25 | Lanzhou, Gansu | 5 times dose Longgensheng | 11.27 |  |  |  |  |  | 452.08 |  |  |  |
| 26 | Lanzhou, Gansu | 5 times dose Longgensheng |  |  | 110.63 |  |  |  | 479.83 |  |  |  |
| 27 | Lanzhou, Gansu | 5 times dose Longgensheng | 18.63 |  | 81.50 |  |  |  | 914.49 |  |  |  |
| 28 | Yunyang, Chongqing | PBZ |  |  |  |  | 388.36 | 90.05 | 64.65 |  |  |  |
| 29 | Yunyang, Chongqing | PBZ |  |  | 87.94 |  | 222.17 | 18.77 | 145.78 |  |  |  |
| 30 | Yunyang, Chongqing | PBZ |  |  | 45.82 | 161.74 | 218.17 | 31.22 | 213.70 |  |  |  |
| 31 | Yunyang, Chongqing | double dose PBZ |  |  | 89.77 |  | 403.05 | 923.45 | 100.81 |  |  |  |
| 32 | Yunyang, Chongqing | double dose PBZ |  |  | 65.81 |  | 35.39 | 386.24 | 199.93 |  |  |  |
| 33 | Yunyang, Chongqing | double dose PBZ |  |  | 157.68 |  | 29.86 | 991.81 | 94.84 |  |  |  |
| 34 | Yunyang, Chongqing | UCZ |  |  | 70.77 | 160.93 | 84.95 | 34.52 |  |  |  |  |
| 35 | Yunyang, Chongqing | UCZ |  | 1883.07 |  | 620.41 | 28.93 |  | 186.04 |  |  |  |
| 36 | Yunyang, Chongqing | UCZ |  |  |  | 747.91 | 89.14 | 315.10 | 142.18 |  |  |  |
| 37 | Longnan, Gansu | Unknown |  |  | 32.90 |  |  |  |  |  |  |  |
| 38 | Longnan, Gansu | Unknown | 454.82 |  | 58.71 |  |  |  | 64.98 |  |  |  |
| 39 | Longnan, Gansu | Unknown | 70.90 |  |  | 43.83 |  |  | 24.80 |  |  |  |
| 40 | Dingxi, Gansu | Unknown | 4.96 |  | 107.38 |  |  |  |  |  |  |  |
| 41 | Dingxi, Gansu | Unknown | 18.03 |  | 167.87 |  |  |  | 602.42 |  |  |  |
| 42 | Dingxi, Gansu | Unknown | 106.80 |  | 29.07 |  |  |  | 116.07 |  |  |  |
| 43 | Dingxi, Gansu | Unknown |  |  |  |  |  |  | 62.76 |  |  |  |
| 44 | Dingxi, Gansu | Unknown | 4.18 |  | 54.00 |  |  |  | 228.46 |  |  |  |
| 45 | Dingxi, Gansu | Unknown | 9.11 |  | 49.89 |  |  |  | 237.39 |  |  |  |
| 46 | Dingxi, Gansu | Unknown | 4.19 |  | 74.01 | 99.54 |  |  | 71.71 |  |  |  |
| 47 | Dingxi, Gansu | Unknown | 1.67 |  |  | 163.96 |  |  |  |  |  |  |
| 48 | Dingxi, Gansu | Unknown |  |  |  | 494.87 |  |  | 139.23 |  |  |  |
| 49 | Shanxi | Unknown |  |  | 71.02 | 12.06 |  | 50.57 |  |  |  |  |
| 50 | Shanxi | Unknown |  |  | 172.05 | 13.40 |  | 6.24 |  | 12.84 |  |  |
| 51 | Shanxi | Unknown |  |  | 236.85 | 4.53 | 324.11 |  |  |  |  |  |
| 52 | Shanxi | Unknown | 84.89 |  | 109.38 | 5.39 | 30.85 |  |  |  |  | 389.98 |
| 53 | Shanxi | Unknown | 16.61 |  | 337.44 | 13.24 | 124.32 |  |  |  |  | 156.66 |
| 54 | Shanxi | Unknown | 11.27 |  | 147.96 | 4.60 |  | 137.71 |  |  |  |  |
| 55 | Shanxi | Unknown | 36.87 | 723.43 | 67.77 | 10.23 |  |  |  |  |  | 176.58 |
| 56 | Shanxi | Unknown |  | 126.49 | 211.56 | 12.76 | 41.22 |  |  |  |  | 188.15 |
| 57 | Shanxi | Unknown | 16.82 |  | 55.77 | 5.34 |  | 36.60 |  |  | 325.62 |  |
| 58 | Shanxi | Unknown | 179.91 |  | 2949.33 | 2.72 |  |  |  |  |  |  |
| 59 | Shanxi | Unknown |  |  | 427.44 | 16.03 |  |  |  |  |  |  |
| 60 | Shanxi | Unknown |  |  | 129.20 | 6.65 |  |  |  |  | 46.33 |  |
| 61 | Shanxi | Unknown |  |  | 9.75 | 4.06 |  |  |  |  |  |  |
| 62 | Shanxi | Unknown |  |  | 218.30 | 10.95 |  |  |  |  | 50.69 |  |
| 63 | Shanxi | Unknown |  |  | 189.83 |  |  | 48.35 |  |  |  |  |
| 64 | Shanxi | Unknown |  |  | 26.84 | 5.28 |  |  |  |  |  | 100.53 |
| 65 | Shanxi | Unknown |  |  | 111.05 | 4.81 |  | 89.13 |  |  |  |  |
| 66 | Shanxi | Unknown |  |  | 119.04 | 5.28 |  |  |  |  |  |  |
| 67 | Shanxi | Unknown |  |  | 19.76 | 4.42 |  |  |  |  |  |  |
| 68 | Shanxi | Unknown |  |  | 227.02 | 20.63 |  |  | 18.57 |  |  |  |
| 69 | Shanxi | Unknown |  |  | 227.02 | 12.85 |  | 6.56 |  |  |  |  |
| 70 | Shanxi | Unknown |  |  | 516.79 | 11.35 |  |  |  |  |  |  |
| 71 | Shanxi | Unknown |  |  | 224.52 | 16.25 |  |  |  |  |  |  |
| 72 | Shanxi | Unknown |  |  | 407.59 | 10.30 |  |  |  |  |  |  |
| 73 | Shanxi | Unknown |  |  | -4.67 |  |  |  |  |  |  |  |
| 74 | Shanxi | Unknown |  |  | 176.16 | 12.40 |  |  |  |  |  |  |
| 75 | Shanxi | Unknown |  |  | 226.52 | 5.04 |  |  |  |  |  |  |
| 76 | Chongqing | Unknown |  | 305.30 | 532.23 | 12.02 |  |  |  |  |  |  |
| 77 | Chongqing | Unknown |  |  | 101.24 |  |  |  |  |  |  |  |
| 78 | Chongqing | Unknown |  |  | 48.92 | 13.55 |  |  | 39.82 |  |  |  |
| 79 | Chongqing | Unknown | 70.90 |  | 248.54 | 7.43 |  |  |  |  |  |  |
| 80 | Chongqing | Unknown |  | 261.46 | 152.29 | 13.29 |  |  | 53.99 |  |  | 238.52 |
| 81 | Chongqing | Unknown |  |  | 147.78 | 3.34 |  |  |  |  |  | 655.89 |
| 82 | Chongqing | Unknown | 18.03 |  | 143.96 | 4.38 |  |  | 66.32 |  |  |  |
| 83 | Chongqing | Unknown | 106.80 |  | -4.67 | 2.80 |  |  |  | 70.36 |  |  |
| 84 | Chongqing | Unknown |  |  | 59.57 |  |  |  |  |  |  |  |
| 85 | Chongqing | Unknown | 11.74 |  | 13.60 |  |  |  |  |  |  |  |
| 86 | Chongqing | Unknown | 30.06 |  | 253.07 |  |  | 52.37 |  |  |  |  |
| 87 | Chongqing | Unknown |  |  | 437.69 |  |  |  |  |  |  |  |
| 88 | Chongqing | Unknown |  |  | 102.27 |  | 79.29 |  |  |  |  |  |
| 89 | Chongqing | Unknown |  |  | 8.20 |  | 39.82 |  |  |  |  |  |
| 90 | Chongqing | Unknown |  |  | 112.71 |  |  |  | 10.85 |  | 50.66 |  |
| 91 | Chongqing | Unknown |  |  | 80.77 | 6.04 |  |  |  |  |  |  |
| 92 | Chongqing | Unknown |  | 42.51 | 232.88 |  |  |  |  |  |  |  |
| 93 | Chongqing | Unknown |  |  | 189.55 |  |  |  |  |  |  | 155.65 |
| 94 | Chongqing | Unknown |  |  | 185.74 |  |  | 68.91 |  |  |  |  |
| 95 | Chongqing | Unknown | 5.02 |  | 253.96 |  |  |  |  |  | 82.669 |  |
| 96 | Chongqing | Unknown |  |  | 134.78 |  |  | 32.20 | 21.99 |  |  |  |
| 97 | Chongqing | Unknown | 76.40 |  | 226.27 |  |  | 92.50 |  |  |  | 222.97 |
| 98 | Chongqing | Unknown |  |  | 366.73 |  | 53.99 |  | 32.32 |  |  |  |
| 99 | Chongqing | Unknown | 7.70 |  | 193.81 |  | 31.44 |  |  |  |  |  |
| 100 | Chongqing | Unknown | 251.21 |  | 144.93 |  |  |  |  |  |  | 115.72 |
| 101 | Chongqing | Unknown |  |  | 68.55 |  |  | 64.12 |  |  |  | 104.93 |
| 102 | Chongqing | Unknown |  |  | 421.10 |  |  |  |  |  |  |  |

Table S6 Components identified in CR extract by UPLC-Q-Orbitrap MS

| NO | Compounds | RT (min) | Adduct ions | *m/z* (detected) | Molecular formula | Calc. MW | Delta mass (ppm) | MS/MS fragments (*m/z*) | Type |
| --- | --- | --- | --- | --- | --- | --- | --- | --- | --- |
| 1 | Arginine^*^ | 0.82 | [M+H]^+^ | 175.118 | C_6_H_14_N_4_O_2_ | 174.1111 | -5.26 | 158.09, 139.00, 114.05, 111.00, 70.06 | Amino acid |
| 2 | Proline^*^ | 0.87 | [M+H]^+^ | 116.0702 | C_5_H_9_NO_2_ | 115.0628 | -3.56 | 70.05 | Amino acid |
| 3 | Adenosine^*^ | 1.50 | [M+H]^+^ | 268.1026 | C_10_H_13_N_5_O_4_ | 267.0962 | -5.11 | 180.87, 136.06, 119.03 | Alkaloids |
| 4 | Guanosine | 1.60 | [M+H]^+^ | 284.0974 | C_10_H_13_N_5_O_5_ | 283.0911 | -5.3 | 152.06, 118.08, 88.08 | Nucleosides |
| 5 | Codonopsinol A | 1.62 | [M+H]^+^ | 270.1322 | C_13_H_19_NO_5_ | 269.1257 | -5.29 | 161.06, 121.06, 90.05, 72.04 | Alkaloids |
| 6 | Codonopsinol | 1.77 | [M+H]^+^ | 284.1477 | C_14_H_21_NO_5_ | 283.1414 | -5.56 | 118.09, 100.08, 88.08, 58.07 | Alkaloids |
| 7 | Codonopsionl C | 1.87 | [M+H]^+^ | 240.1217 | C_12_H_17_NO_4_ | 239.1152 | -5.55 | 222.11, 175.07, 131.04, 121.06 | Alkaloids |
| 8 | Phenylalanine^*^ | 2.80 | [M+H]^+^ | 166.0854 | C_9_H_11_NO_2_ | 165.0784 | -5.33 | 120.08, 107.05, 79.05 | Amino acid |
| 9 | Codonopiloside A | 3.46 | [M+H]^+^ | 416.1896 | C_19_H_29_NO_9_ | 415.1836 | -4.63 | 161.06, 127.04, 104.07, 85.03, 74.06 | Alkaloids |
| 10 | Codonopsinol B | 3.61 | [M+H]^+^ | 254.1376 | C_13_H_19_NO_4_ | 253.1309 | -4.25 | 236.12, 205.08, 186.95, 158.96, 128.95 | Alkaloids |
| 11 | Protocatechuic acid | 3.63 | [M+H]^+^ | 153.0177 | C_7_H_6_O_4_ | 154.0272 | -3.5 | 109.02 | Organic acid |
| 12 | Vanillic acid | 3.69 | [M+H]^+^ | 167.0328 | C_8_H_8_O_4_ | 168.0428 | -6.02 | 123.04, 93.03, 65.04 | Organic acid |
| 13 | Codonopyrrolidium B | 3.83 | M^+^ | 268.1528 | C_14_H_22_NO_4_ | 268.1543 | -5.49 | 161.06, 118.08, 100.08, 88.07 | Alkaloids |
| 14 | 3'-hydroxycodonopyrrolidiumC | 4.34 | [M+H]^+^ | 367.1957 | C_19_H_28_NO_6_ | 366.1911 | -8.82 | 266.13, 221.08, 177.05, 137.05, 100.07, 88.0756 | Alkaloids |
| 15 | Trytophan fructoside | 4.77 | [M+H]^+^ | 365.1344 | C_17_H_22_N_2_O_7_ | 366.1432 | 0.20 | 275.11, 203.08, 116.05 | Amino acid |
| 16 | 6-methyoxy-4-formyl quinoline | 4.98 | [M+H]^+^ | 188.0694 | C_11_H_9_NO_2_ | 187.0627 | -5.93 | 170.06, 146.06, 118.06, 90.95 | Alkaloids |
| 17 | Tryptophan^*^ | 4.98 | [M+H]^+^ | 205.0960 | C_11_H_12_N_2_O_2_ | 204.0893 | -5.65 | 188.07, 170.06, 146.06, 144.08, 118.06 | Amino acid |
| 18 | 3-Indoleacrylic acid | 4.98 | [M+H]^+^ | 188.0698 | C_11_H_9_NO_2_ | 187.0627 | -4.15 | 159.96, 146.05, 128.95, 118.06 | Organic acid |
| 19 | Caffeic acid dihexoside I | 5.43 | [M-H]^-^ | 503.1396 | C_21_H_28_O_14_ | 504.1484 | 0.14 | 341.11, 179.05, 119.04 | Organic acid |
| 20 | 3-O-Caffeoylquinic acid | 6.92 | [M+H]^+^ | 355.1006 | C_16_H_18_O_9_ | 354.0954 | -4.89 | 163.03, 135.04, 117.02, 89.03 | Organic acid |
| 21 | Chlorogenic acid | 7.01 | [M+H]^+^ | 355.1005 | C_16_H_18_O_9_ | 354.0956 | -5.23 | 191.06, 146.94, 102.95 | Organic acid |
| 22 | Tangshenyne A | 8.78 | [M+HCOO]^-^ | 457.1706 | C_20_H_28_O_9_ | 412.1739 | 0.39 | 435.18, 412.55, 391.20, 264.55 | Polyacetylene |
| 23 | Geniposide | 8.80 | [M-H]^-^ | 387.1288 | C_17_H_24_O_10_ | 388.1375 | 0.61 | 387.13, 207.05, 179.05, 161.04, 119.03, 102.02 | Others |
| 24 | Anti-coumaric acid | 9.55 | [M-H]^-^ | 165.0538 | C_9_H_8_O_3_ | 164.0479 | -4.97 | 147.04, 119.05, 103.05, 91.05 | Organic acid |
| 25 | 3'-hydroxycodonopyrrolidium C | 9.55 | [M+H]^+^ | 366.1891 | C_19_H_27_NO_6_ | 365.1844 | -5.44 | 170.11, 137.06, 100.08, 88.08, 83.05 | Alkaloids |
| 26 | Lobetyolinin Isomer | 9.74 | [M+HCOO]^-^ | 603.2289 | C_26_H_38_O_13_ | 558.2317 | 0.84 | 233.11, 218.86, 215.11, 133.07, 119.03, 101.02 | Polyacetylene |
| 27 | Tangshenoside I | 9.81 | [M-H]^-^ | 677.2283 | C_29_H_42_O_18_ | 678.2377 | -0.87 | 396.91, 276.09, 217.09, 99.04 | Phenylpropanoid |
| 28 | P-coumaricacidglucoside | 9.85 | [M+H]^+^ | 327.1058 | C_15_H_18_O_8_ | 326.1007 | -5.03 | 219.82, 161.04, 119.05, 91.05, 71.01 | Organic acid |
| 29 | 3'-hydroxycodonopyrrolidium F | 9.94 | [M+H]^+^ | 368.2051 | C_19_H_29_NO_6_ | 367.2000 | -4.41 | 172.13, 137.06, 100.08, 88.08, 85.06 | Alkaloids |
| 30 | 3'-hydroxycodonopyrrolidium G | 10.05 | [M+H]^+^ | 368.2052 | C_19_H_29_NO_6_ | 367.2000 | -4.32 | 172.13, 137.06, 100.08, 88.08, 85.06 | Alkaloids |
| 31 | Tangshenoside V | 10.27 | [M-H]^-^ | 469.1331 | C_21_H_26_O_12_ | 352.2118 | -1.86 | 325.09, 163.03, 119.04 | Phenylpropanoid |
| 32 | Lobetyolinin Isomer | 10.38 | [M+HCOO]^-^ | 603.2288 | C_26_H_38_O_13_ | 558.2317 | 0.78 | 233.11, 218.86, 215.11, 133.07, 119.03, 101.02 | Polyacetylene |
| 33 | 3'-hydroxycodonopyrrolidium D | 10.50 | [M+H]^+^ | 368.2048 | C_19_H_29_NO_6_ | 367.2000 | -5.33 | 172.13, 137.06, 100.08, 88.08, 85.06 | Alkaloids |
| 34 | Hexyl-β-D-glucopyranosyl-(1→6)-β-D-glucopyranosid | 10.50 | [M-H]^-^ | 425.2017 | C_18_H_34_O_11_ | 426.2096 | -0.05 | 263.15, 161.04, 159.02, 101.02 | Others |
| 35 | TangshenosideV or Isomer | 10.56 | [M-H]^-^ | 469.1342 | C_21_H_26_O_12_ | 352.2118 | 0.23 | 325.09, 163.03, 119.04 | Phenylpropanoid |
| 36 | 3'-hydroxycodonopyrrolidium H | 10.66 | [M+H]^+^ | 368.2048 | C_19_H_29_NO_6_ | 367.2000 | -5.06 | 172.13, 137.06, 100.08, 88.08, 85.06 | Alkaloids |
| 37 | Codonopyrrolidium A | 10.84 | [M+H]^+^ | 350.1948 | C_19_H_27_NO_5_ | 349.1894 | -1.44 | 220.13, 161.06, 121.06, 100.08, 88.08 | Alkaloids |
| 38 | Lobetyolinin Isomer | 11.02 | [M+HCOO]^-^ | 603.2289 | C_26_H_38_O_13_ | 558.2317 | 0.88 | 233.11, 218.86, 215.11, 133.07, 119.03, 101.02 | Polyacetylene |
| 39 | Codonopyrrolidium F | 11.24 | [M+H]^+^ | 352.2101 | C_19_H_29_NO_5_ | 351.2040 | -1.68 | 219.12, 187.07, 161.06, 121.06, 98.06, 88.08 | Alkaloids |
| 40 | Lobetyolinin | 11.24 | [M+HCOO]^-^ | 603.2285 | C_26_H_38_O_13_ | 558.2317 | 0.17 | 221.07, 161.04, 125.02, 89.02, 59.01 | Polyacetylene |
| 41 | TangshenosideV or Isomer | 11.28 | [M-H]^-^ | 469.1339 | C_21_H_26_O_12_ | 352.2118 | -0.36 | 325.09, 163.03, 119.04 |  |
| 42 | Codonopilodiynoside H | 11.28 | [M+HCOO]^-^ | 605.2444 | C_26_H_38_O_13_ | 558.2307 | 0.633 | 435.87, 353.81, 221.07, 161.04, 113.02 | Polyacetylene |
| 43 | Codonopyrrolidium G | 11.38 | [M+H]^+^ | 352.2101 | C_19_H_29_NO_5_ | 351.204 | -1.68 | 219.12, 187.07, 161.06, 121.06, 98.06, 88.08 | Alkaloids |
| 44 | Lobetyolinin Isomer | 11.52 | [M+HCOO]^-^ | 603.2287 | C_26_H_38_O_13_ | 558.2317 | 0.58 | 221.07, 161.04, 125.02, 89.02, 59.01 | Polyacetylene |
| 45 | Azelaic acid | 11.69 | [M+H]^+^ | 189.1112 | C_9_H_16_O_4_ | 188.1043 | -1.02 | 158.98, 125.09, 116.92 | Organic acid |
| 46 | Neokurarinol | 11.72 | [M-H]^-^ | 467.2119 | C_27_H_32_O_7_ | 468.2142 | 3.5 | 309.15, 235.92, 207.92, 158.97, 116.92 | Alkaloids |
| 47 | Codonopyrrolidium D | 11.77 | [M+H]^+^ | 352.2102 | C_19_H_29_NO_5_ | 351.2040 | -1.69 | 219.12, 187.07, 161.06, 121.06, 98.06, 88.08 | Alkaloids |
| 48 | Ferulic acid | 11.94 | [M-H]^-^ | 193.0491 | C_10_H_10_O_4_ | 194.0585 | -0.47 | 178.02, 149.06, 134.03 | Organic acid |
| 49 | Codonopyrrolidium E | 11.96 | [M+H]^+^ | 352.2102 | C_19_H_29_NO_5_ | 351.2040 | -1.69 | 219.12, 187.07, 161.06, 121.06, 98.06, 88.08 | Alkaloids |
| 50 | Lobetyol | 12.22 | M+H-H_2_O | 217.1212 | C_14_H_18_O_3_ | 234.1261 | -5.23 | 217.12, 199.11, 128.06, 93.0333 | Polyacetylene |
| 51 | Lobetyolin^*^ | 12.23 | [M+HCOO]^-^ | 441.1754 | C_20_H_28_O_8_ | 396.1778 | -0.23 | 398.32, 233.28, 159.08, 143.07, 125.06 | Polyacetylene |
| 52 | Codonopilodiynoside D | 12.25 | [M+HCOO]^-^ | 443.1918 | C_20_H_30_O_8_ | 398.1935 | 1.34 | 305.09, 174.81, 161.04, 119.03, 113.02, 101.02 | Polyacetylene |
| 53 | TangshenosideVI | 12.48 | [M-H]^-^ | 451.1231 | C_21_H_24_O_11_ | 452.1313 | -0.90 | 407.13, 325.08, 163.04, 119.05 | Phenylpropanoid |
| 54 | α-D-furantohexyl glycoside | 12.89 | [M-H]^-^ | 263.1490 | C_12_H_24_O_6_ | 264.1578 | 0.17 | 161.04, 119.05, 101.02, 59.01 | Others |
| 55 | TangshenosideVI Isomer | 13.08 | [M-H]^-^ | 451.1232 | C_21_H_24_O_11_ | 452.1313 | -0.64 | 407.13, 325.08, 163.04, 119.05 | Phenylpropanoid |
| 56 | Tangshenoside VI Isomer | 13.32 | [M-H]^-^ | 451.1233 | C_21_H_24_O_11_ | 452.1313 | -0.36 | 407.13, 325.08, 163.04, 119.05 | Phenylpropanoid |
| 57 | Atractylenolide III | 18.01 | [M+H]^+^ | 249.1475 | C_15_H_20_O_3_ | 248.1407 | -4.07 | 231.13, 142.15, 100.11, 94.04 | Terpenoids |
| 58 | Atraetylenolide I | 18.14 | [M+H]^+^ | 231.1368 | C_15_H_18_O_2_ | 230.1301 | -4.96 | 185.12, 175.07, 143.06, 119.09, 105.07 | Terpenoids |
| 59 | Atraetylenolide II Isomer | 19.22 | [M+H]^+^ | 233.1524 | C_15_H_20_O_2_ | 232.1478 | -5.00 | 215.14, 142.15, 100.11 | Terpenoids |
| 60 | Atractylenolide Ⅱ | 19.34 | [M+H]^+^ | 233.1528 | C_15_H_20_O_2_ | 232.1478 | -3.38 | 215.14, 142.15, 100.11 | Terpenoids |
| 61 | Coronaricacid | 19.91 | [M-H]^-^ | 295.2273 | C_18_H_32_O_3_ | 296.2346 | 1.89 | 265.14, 116.92 | Organic acid |

Abbreviations: ^*^ Identified with authentic standards

Table S7 LC-HR-MS/MS methodological investigation results

| NO. | Ion mode | RT（min） | *m/z* | Precision (RSD)/% | | Repeatability (RSD)/% | | Stability (RSD)/% | |
| --- | --- | --- | --- | --- | --- | --- | --- | --- | --- |
|  |  |  |  | RT | Area | RT | Area | RT | Area |
| 1 | Positive | 1.14 | 280.138 | 0.41 | 5.05 | 0.27 | 6.56 | 0.23 | 4.92 |
| 2 | Negative | 4.51 | 205.840 | 0.42 | 6.53 | 0.31 | 6.93 | 0.28 | 6.76 |
| 3 | Positive | 7.62 | 341.138 | 0.26 | 5.21 | 0.38 | 5.25 | 0.38 | 6.16 |
| 4 | Negative | 9.05 | 97.957 | 0.34 | 5.76 | 0.29 | 3.87 | 0.46 | 2.54 |
| 5 | Negative | 11.05 | 515.118 | 0.25 | 3.45 | 0.29 | 3.14 | 0.35 | 3.11 |
| 6 | Positive | 13.79 | 313.143 | 0.32 | 4.33 | 0.24 | 3.68 | 0.25 | 3.73 |
| 7 | Negative | 16.40 | 297.134 | 0.26 | 3.55 | 0.29 | 3.19 | 0.42 | 3.25 |
| 8 | Positive | 18.95 | 350.175 | 0.24 | 5.88 | 0.36 | 6.67 | 0.22 | 7.15 |
| 9 | Negative | 19.25 | 264.858 | 0.23 | 5.24 | 0.28 | 6.66 | 0.31 | 6.78 |
| 10 | Positive | 19.87 | 470.251 | 0.21 | 4.21 | 0.23 | 4.56 | 0.35 | 4.20 |

Table S8 The changes in the differential metabolites for Pingshun

| NO | Compounds | Type | VIP | *P*-value | Fold Change | Type |
| --- | --- | --- | --- | --- | --- | --- |
| 1 | Arginine | Amino acid | 2.02 | 6.6E-06 | 1.87 | up |
| 2 | Adenosine | Alkaloids | 1.82 | 0.015044 | 0.89 | down |
| 3 | Codonopsinol | Alkaloids | 1.82 | 0.000676 | 0.33 | down |
| 4 | Codonopiloside A | Alkaloids | 1.08 | 0.038026 | 0.53 | down |
| 5 | Protocatechuic acid | Organic acid | 1.27 | 0.035487 | 0.81 | down |
| 6 | Codonopyrrolidium B | Alkaloids | 1.60 | 0.028898 | 0.74 | down |
| 7 | 3'-hydroxycodonopyrrolidiumC | Alkaloids | 1.69 | 0.004009 | 2.33 | up |
| 8 | Tryptophan | Amino acid | 1.21 | 0.034233 | 1.27 | up |
| 9 | 3'-hydroxycodonopyrrolidium C | Alkaloids | 1.29 | 0.041283 | 0.56 | down |
| 10 | Tangshenoside I | Phenylpropanoid | 1.22 | 0.025892 | 0.85 | down |
| 11 | 3'-hydroxycodonopyrrolidium F | Alkaloids | 1.72 | 0.038719 | 0.25 | down |
| 12 | 3'-hydroxycodonopyrrolidium G | Alkaloids | 1.87 | 0.005183 | 0.34 | down |
| 13 | 3'-hydroxycodonopyrrolidium D | Alkaloids | 1.74 | 0.001689 | 0.33 | down |
| 14 | 3'-hydroxycodonopyrrolidium H | Alkaloids | 1.69 | 0.005018 | 0.29 | down |
| 15 | Codonopyrrolidium A | Alkaloids | 2.13 | 0.003265 | 0.57 | down |
| 16 | Codonopyrrolidium F | Alkaloids | 1.54 | 0.027656 | 0.42 | down |
| 17 | Lobetyolinin | Polyacetylene | 1.35 | 0.043132 | 1.40 | up |
| 18 | Codonopyrrolidium G | Alkaloids | 2.09 | 0.020619 | 0.47 | down |
| 19 | Neokurarinol | Alkaloids | 1.24 | 0.020744 | 0.67 | down |
| 20 | Codonopyrrolidium D | Alkaloids | 1.96 | 0.011849 | 0.68 | down |
| 21 | Codonopyrrolidium E | Alkaloids | 1.80 | 0.013725 | 0.64 | down |
| 22 | Atractylenolide III | Terpenoids | 1.93 | 0.008871 | 0.66 | down |
| 23 | Atraetylenolide II Isomer | Terpenoids | 2.37 | 0.002676 | 0.55 | down |
| 24 | Atractylenolide Ⅱ | Terpenoids | 2.28 | 0.01071 | 0.56 | down |
| 25 | Coronaricacid | Organic acid | 1.16 | 0.03672 | 0.68 | down |

Table S9 The changes in the differential metabolites for Huguan

| NO | Compounds | Type | VIP | *P*-value | Fold Change | Type |
| --- | --- | --- | --- | --- | --- | --- |
| 1 | Arginine | Amino acid | 1.99 | 7.22E-05 | 1.64 | up |
| 2 | Tangshenoside I | Phenylpropanoid | 1.21 | 0.026466 | 0.85 | down |
| 3 | Lobetyolinin | Polyacetylene | 1.35 | 0.042741 | 1.40 | up |
| 4 | Tryptophan | Amino acid | 1.76 | 0.00237 | 1.48 | up |
| 5 | Neokurarinol | Alkaloids | 1.24 | 0.020744 | 0.67 | down |
| 6 | Hexyl-β-D-glucopyranosyl-(1→6)-β-D-glucopyranosid | Others | 1.21 | 0.020815 | 0.63 | down |
| 7 | Protocatechuic acid | Organic acid | 1.27 | 0.035487 | 0.81 | down |
| 8 | 3'-hydroxycodonopyrrolidium G | Alkaloids | 1.93 | 0.002178 | 0.42 | down |
| 9 | Codonopsinol | Alkaloids | 2.27 | 3.53E-05 | 0.48 | down |
| 10 | 3'-hydroxycodonopyrrolidium C | Alkaloids | 1.39 | 0.027118 | 0.66 | down |
| 11 | 3'-hydroxycodonopyrrolidium H | Alkaloids | 1.84 | 0.00335 | 0.36 | down |
| 12 | Coronaricacid | Organic acid | 1.16 | 0.03672 | 0.68 | down |
| 13 | 3'-hydroxycodonopyrrolidiumC | Alkaloids | 1.73 | 0.004625 | 2.06 | up |
| 14 | 3'-hydroxycodonopyrrolidium D | Alkaloids | 1.88 | 0.000956 | 0.42 | down |
| 15 | Codonopiloside A | Alkaloids | 1.08 | 0.038026 | 0.53 | down |
| 16 | Codonopyrrolidium B | Alkaloids | 2.24 | 4.83E-05 | 0.52 | down |
| 17 | Codonopyrrolidium E | Alkaloids | 1.64 | 0.007419 | 0.38 | down |
| 18 | Proline | Amino acid | 1.67 | 0.003686 | 1.22 | up |
| 19 | Codonopyrrolidium D | Alkaloids | 1.48 | 0.013943 | 0.55 | down |
| 20 | Codonopyrrolidium G | Alkaloids | 1.83 | 0.001176 | 0.50 | down |
| 21 | Codonopsinol B | Alkaloids | 1.30 | 0.04929 | 0.56 | down |
| 22 | Codonopyrrolidium A | Alkaloids | 1.44 | 0.016885 | 0.70 | down |
| 23 | 6-methyoxy-4-formyl quinoline | Alkaloids | 1.69 | 0.003521 | 1.41 | up |
| 24 | 3-Indoleacrylic acid | Organic acid | 1.69 | 0.003521 | 1.41 | up |
| 25 | Codonopyrrolidium F | Alkaloids | 1.53 | 0.011158 | 0.47 | down |
| 26 | Atractylenolide Ⅱ | Terpenoids | 1.88 | 0.00051 | 0.59 | down |
| 27 | Atractylenolide III | Terpenoids | 1.84 | 0.001009 | 0.49 | down |
| 28 | Atraetylenolide II Isomer | Terpenoids | 2.08 | 5.28E-05 | 0.46 | down |
| 29 | Atraetylenolide I | Terpenoids | 1.30 | 0.042708 | 0.75 | down |
